# Contribution of m5C RNA Modification-Related Genes to Prognosis and Immunotherapy Prediction in Patients with Ovarian Cancer

**DOI:** 10.1155/2023/1400267

**Published:** 2023-11-13

**Authors:** Yibin Liu, Shouze Liu, Lu Yan, Qianqian Zhang, Wenhua Liu, Xianghua Huang, Shikai Liu

**Affiliations:** ^1^Department of Gynecology, The Second Hospital of Hebei Medical University, 215 Heping West Road, Shijiazhuang, Hebei 050011, China; ^2^Department of Gynecology III, Cangzhou Central Hospital, Cangzhou, Hebei 061000, China; ^3^Department of Gynecology and Obstetrics, Beijing Tsinghua Changgung Hospital, Beijing 102218, China; ^4^Department of Pain, Cangzhou Hospital of Integrated TCM-WM Hebei, Cangzhou, Hebei 061001, China

## Abstract

**Background:**

5-Methylcytosine (m5C) RNA modification is closely implicated in the occurrence of a variety of cancers. Here, we established a novel prognostic signature for ovarian cancer (OC) patients based on m5C RNA modification-related genes and explored the correlation between these genes with the tumor immune microenvironment.

**Methods:**

Methylated-RNA immunoprecipitation sequencing helped us to identify candidate genes related to m5C RNA modification at first. Based on TCGA database, we screened the differentially expressed candidate genes related to the prognosis and constructed a prognostic model using LASSO Cox regression analyses. Notably, the accuracy of the model was evaluated by Kaplan–Meier analysis and receiver operator characteristic curves. Independent prognostic risk factors were investigated by Cox proportional hazard model. Furthermore, we also analyzed the biological functions and pathways involved in the signature. Finally, the immune response of the model was visualized in great detail.

**Results:**

Totally, 2,493 candidate genes proved to be involved in m5C modification of RNA for OC. We developed a signature with prognostic value consisting of six m5C RNA modification-related genes. Specially, samples have been split into two cohorts with low- and high-risk scores according to the model, in which the low-risk OC patients exhibited dramatically better overall survival time than those with high-risk scores. Besides, not only was this model a prognostic factor independent of other clinical characteristics but it predicted the intensity of the immune response in OC. Significantly, the accuracy and availability of the signature were verified by ICGC database.

**Conclusions:**

Our study bridged the gap between m5C RNA modification and the prognosis of OC and was expected to provide an effective breakthrough for immunotherapy in OC patients.

## 1. Introduction

As one of the most common gynecological malignant tumors, ovarian cancer (OC) kills more than 200,000 women annually [1]. The high-mortality rate of OC is closely related to its asymptomatic nature, with a 5-year survival rate of less than 50% [2]. Cytoreductive surgery combined with chemotherapy remains the gold standard of treatment for OC. However, chemotherapy resistance followed by intraperitoneal dissemination still leads to unpredictable deaths, possibly due to the neglect of tumor cell heterogeneity, thus treating it as a single disease [3]. Therefore, personalized treatment seems to be a feasible strategy for improving the quality of prognosis in the patients with OC.

Being considered as an emerging gene expression regulation method, posttranscriptional RNA modifications are involved in the development of a range of human diseases, including cancer, which may develop to be an ideal target for cancer therapy [4, 5]. RNA epigenetic modifications mainly involve N1-methyladenosine (m1A), pseudouridine (*ψ*), 5-methylcytosine (m5C), 5-hydroxymethylcytosine (hm5C), N6-methyladenosine (m6A), and N7-methylguanosine (m7G) [5]. Characterized by the attachment of a methyl group to the fifth carbon atom in cytosine residues, RNA m5C participates in the metabolism and structural stability of RNA and the recognition of transfer RNAs (tRNA), which allows dynamic regulation of cellular responses to adapt to rapidly changing microenvironments. For example, adaptation to the chemotherapeutic drugs plays a vital role in ensuring the survival of tumor cells [6, 7], suggesting an affinity between RNA m5C and cancer cells. Recent studies have shown that RNA m5C performs well in predicting prognosis and immune infiltration characteristics in triple-negative breast cancer [8], cervical cancer [9], hepatocellular carcinoma [10], colorectal cancer [11, 12], clear cell renal cell carcinoma [13], and lung squamous cell carcinoma and adenocarcinoma [14, 15], the role of RNA m5C in OC prognosis remains to be elucidated.

In the present study, we attempted to expand our understanding of the effects of RNA m5C-related genes in the affecting of overall survival (OS) in OC. In addition, efforts have been made to reveal the potential role of these genes in the changes in the immune microenvironment and chemosensitivity of OC, which might help in improving treatment strategies.

## 2. Materials and Methods

### 2.1. Sample Collection and Ethical Approval

Three pairs of high-grade serous ovarian cancer (HGSOC) samples and corresponding adjacent tissues were obtained from the Second Hospital of Hebei Medical University, and all tissues were histopathologically confirmed by pathologists. After surgery, the HGSOC samples and adjacent normal tissues were immediately stored at −80°C for further use. This study was approved by the Ethics Committee and Institutional Review Board of the Second Hospital of Hebei Medical University. Written informed consent was obtained from all participants before the study began. This study was conducted in accordance with the Declaration of Helsinki.

### 2.2. Detection of m5C RNA Modification-Related Genes

First, we used TRIzol reagent (Invitrogen Corporation, Carlsbad, CA) to extract RNA. The m5C-methylated mRNA fragments were enriched by immunoprecipitation with an anti-m5C antibody (Synaptic Systems). The NEBNext Ultra II Directional RNA Library Prep Kit (New England Biolabs, Inc.) was used to construct the cDNA library, whose quality and purity were evaluated using the Agilent Bioanalyzer 2100 system (Agilent Technologies, Inc., USA). Sequentially, DCC and MACS (v1.4.2) were applied to identify circular RNA (circRNA) peaks and methylated m5C peaks on the circRNAs. Differentially, m5C methylation peaks were filtered with a fold change >2 or < 0.5 (*p*-value < 0.00001) using diffReps software. The circRNA region where the m5C peak was located was calculated for the two cohorts. Statistical significance was set at *p* < 0.05. Finally, we combined transcriptome and methylation analyses to identify the effects of methylation on transcriptional expression.

### 2.3. Original Data Acquisition

The RNA-seq data from 88 normal samples and 379 OC tissues were extracted from the Genotype-Tissue Expression (GTEx) database (https://xenabrowser.net/) and The Cancer Genome Atlas (TCGA) (https://portal.gdc.cancer.gov/), respectively. The corresponding clinical information was also downloaded. Then we unified the two sets of expression data from different sources using “limma” package in R 4.1.1 and got normalized datasets [16]. We collected the patients' informed consent for the publication of the data used in the experiment. Additionally, the public materials involved in this study were downloaded free of charge from the public database; therefore, ethical review and approval are optional from the Ethics Committee.

### 2.4. Data Normalization

For the purpose of integrating the expression data from GTEx and TCGA database, we utlized the batch normalization to correct unwanted technical variation. At first, log2(*x* + 0.001) transformed RNA-seq FPKM values of ovary tissue were extracted from GTEx database. In the meanwhile, we obtained the RNA-seq FPKM data of TCGA-OV patients. Then, these expression data from both databases were log2(*x* + 1) transformed. Finally, batch normalization was performed across abovementioned data by the function named normalizeBetweenArrays in “limma” package in R 4.1.1 [17].

### 2.5. Calculation of Differentially Expressed Genes (DEGs) Related to Survival Time

We performed the DEGs screening process using R with the filtering criteria of false discovery rate (FDR) < 0.01 and |log2 FC|≥ 2. *p*-Values were adjusted to control for FDR using the Benjamini–Hochberg method, which can enhance the reliability of the results [18]. At the same time, univariate Cox regression analysis was applied to determine the target genes valuable for prognosis. Finally, the core genes in this signature were generated after intersection of the two gene sets.

### 2.6. Development of the Prognostic Model

With the aid of Lasso-penalized Cox regression analysis, we calculated the risk coefficient (coef) of each m5C-related gene and determined each sample's risk score combined with gene expression. Each OC patient was assigned a risk score, which = gene1 (coef  ^*∗*^ expression) + gene2 (coef  ^*∗*^ expression) + .. gene7 (coef  ^*∗*^ expression). As such, we built an m5C-related model in the TCGA cohort to predict the risk characteristics of OC patients, in which those with a risk score less than the median value were recognized as the low-risk group, whereas the others were recognized as the high-risk group. To evaluate the accuracy of this signature in prognostic value, we plotted Kaplan–Meier (K–M) survival curves and time-dependent receptor operating characteristic (ROC) curves using “survival” and “timeROC” package in R. Similarly, the median score in TCGA was used as a cutoff value to distinguish high- and low-risk groups in The International Cancer Genome Consortium (ICGC) (https://dcc.icgc.org/), and survival differences between two risk statuses were explored further following above steps.

### 2.7. Evaluation for the Performance of the Signature

Principal component analysis (PCA) and t-distributed Stochastic Neighbor Embedding (t-SNE) were employed to investigate whether OC patients could be significantly distinguished based on the m5C-related risk scores. Multivariate Cox analysis was used to determine whether m5C-related risk scores could be an independent risk factor affecting the prognosis of patients was figured out using multivariate Cox analysis. After that, we constructed a nomogram to predict the 1-, 3-, and 5-year OS utilizing “rms” package in R.

### 2.8. Biological Function and Pathway Enrichment Analysis

According to our previous method [19], the gene sets of differentially expressed genes between the two risk cohorts were prepared for the subsequent steps followed by the thresholds:*p*=0.05 and logFC = 1. We used the Kyoto Encyclopedia of Genes and Genomes (KEGG) and gene ontology (GO) analyses to label different signaling pathways and functions, respectively. The annotations were revealed using package “ggplot2” package in R.

### 2.9. Immune Infiltration Exploration

Discrepancies in the abundance of immunocytes and immune-related functions between low- and high-risk populations were assessed using single-sample gene set enrichment analysis (ssGSEA). Of note, 13 immunocyte types and 13 immunological functions were involved in the quantification of immune infiltration. And we controlled the entire process via R package “limma,” “ggpubr,” and “reshape2.”

### 2.10. Drug Sensitivity Prediction

Combining the m5C RNA modification-related genes with drug sensitivity, we assessed the therapeutic response to chemotherapy for OC patients in our signature, which was reflected by half-maximal inhibitory concentration (IC_50_) values. To complete the whole procedure, “pRRophetic” package in R was applied in this prediction.

## 3. Results

### 3.1. Generation of Model Genes in OC

**Figure 1:**
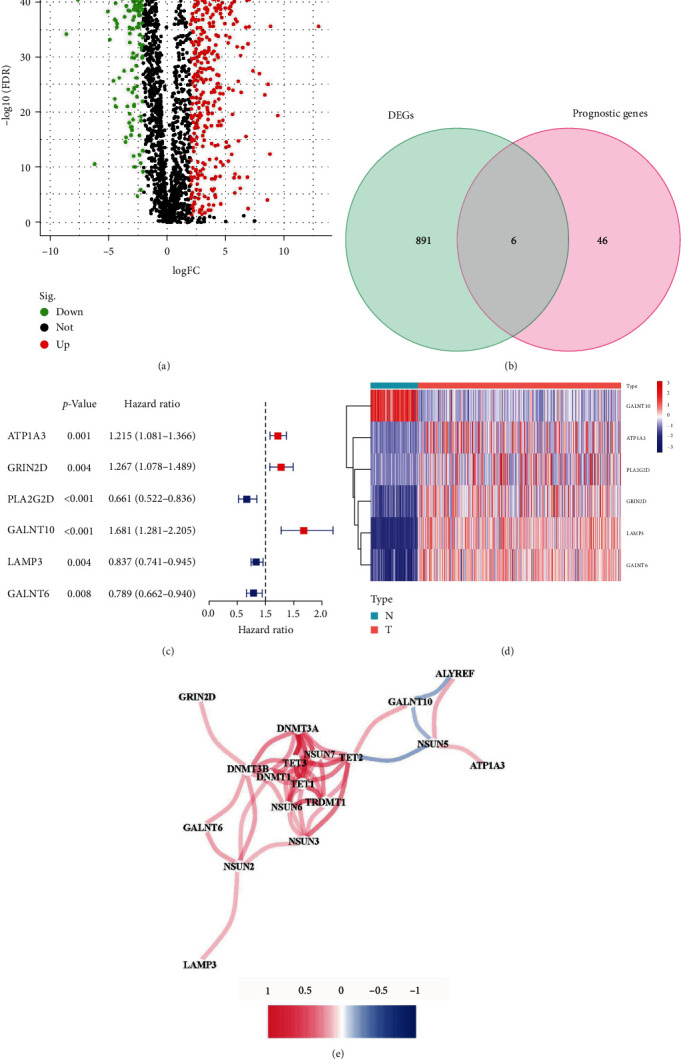
Generation and characteristics of model genes. (a) Green and red dots: genes of significant differentially expressed (|log2 FC|≥ 2 with false discovery rate (FDR) < 0.01); black dots: normally expressed genes. (b) The Venn plot for core genes. (c) The forest plot of core genes. (d) The heat map for expression levels of core genes. (e) The correlation network between core genes and RNA m5C regulators. Blue lines represent negative correlation; red lines represent positive correlation.

Conjoint analysis of transcriptomics and methylome data captured 2,493 m5C RNA modification-related genes from the prophase experiment (*Supplementary 1*), of which 897 were calculated as DEGs between tumor and normal samples in TCGA (Figure 1(a)) and 52 were identified to be significantly correlated with survival time (*Supplementary 2*). Finally, six genes remained (ATP1A3, GRIN2D, PLA2G2D, GALNT10, LAMP3, and GALNT6) after the intersection of the two gene sets (Figure 1(b)). The details are provided in a forest plot and heat map (Figures 1(c) and 1(d)).

### 3.2. Association Analysis between Hub Genes and m5C RNA Methylation Regulators

By means of “igraph” and “reshape2” packages in R, the correlation network captured the features of intimate connectivity among the candidate genes (Figure 1(e)). Significantly, ATP1A3, GRIN2D, LAMP3, and GALNT6 were positively correlated with m5C writers. GALNT10 was positively correlated with an eraser but negatively associated with the writer and reader genes. As shown in *Supplementary 3*, there is a weak positive correlation between PLA2G2D and the regulator of writers [20].

### 3.3. Construction and Evaluation of m5C RNA Modification-Related Signature in OC

**Figure 2:**
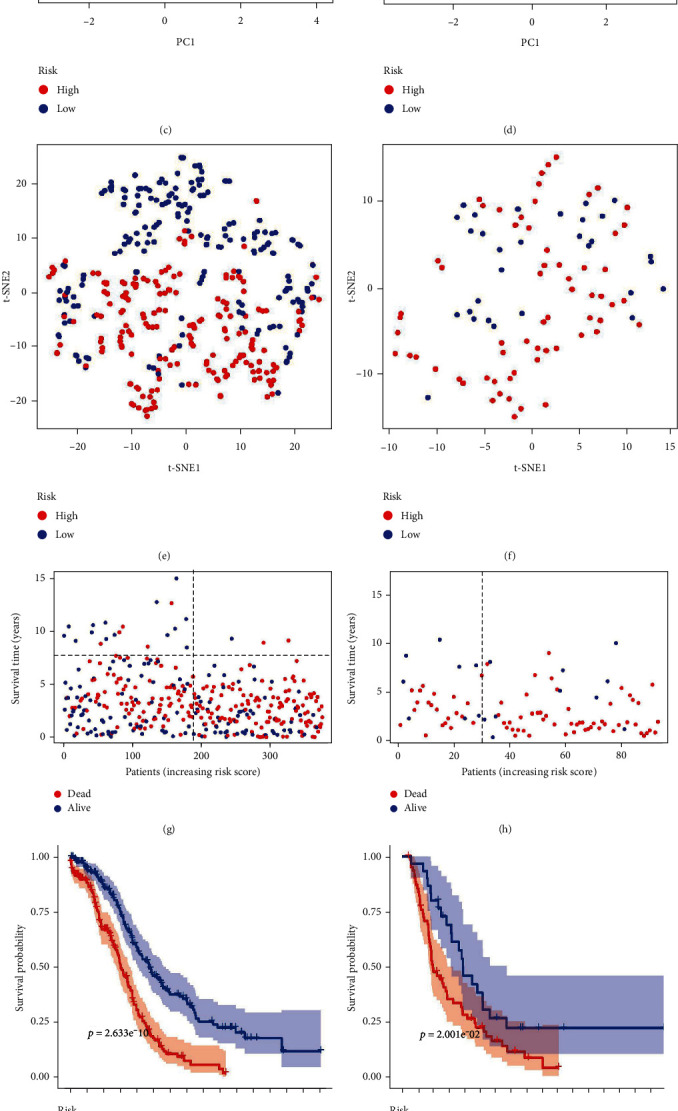
Prognostic signature model structuring and validating. (a, b) The risk scores and distribution of patients from TCGA (a) and ICGC (b). (c, d) PCA plots of the training (c) and testing cohorts (d). (e, f) Results of t-SNE analysis in the training (e) and testing cohorts (f). (g, h) Survival status distribution of patients from TCGA (g) and ICGC (h). (i, j) Kaplan–Meier curves for the training (i) and testing groups (j). (k, l) the ROC curves in the training (k) and the testing sets (l).

By listing the coef values of the six genes (Table 1), we can use the formula mentioned above to compute the risk score of each sample in both training TCGA (Figure 2(a)) and testing ICGC (Figure 2(b)). We noticed distributional differences in OC patients, characterized by the risk scores in PCA and t-NSE (Figure 2(c)–2(f)). In addition, as the risk score increased, the spread of patients shifted toward a decreased survival time and higher mortality (Figures 2(g) and 2(h)). And we got to know from K–M survival curves that patients with low-risk scores took on significantly better survival outcomes than the others: training cohort (*p* = 2.633e^−10^) (Figure 2(i)) and testing cohort (*p* = 2.001e^−02^) (Figure 2(j)). Crucially, it was deniable that the ROC curves showed an accurate predictive capability of this signature in 1-, 2- and 3- years OS with high-area under the curve (AUC) values. The AUC were 0.635, 0.693, and 0.670 in the training group and 0.721, 0.700, and 0.661 in the testing group, respectively (Figures 2(k) and 2(l)). Based on these results, our model demonstrated robust predictive performance for OS in general OC patients.

### 3.4. Comprehensive Investigation of Risk Factors Affecting Survival in OC Patients

**Figure 3:**
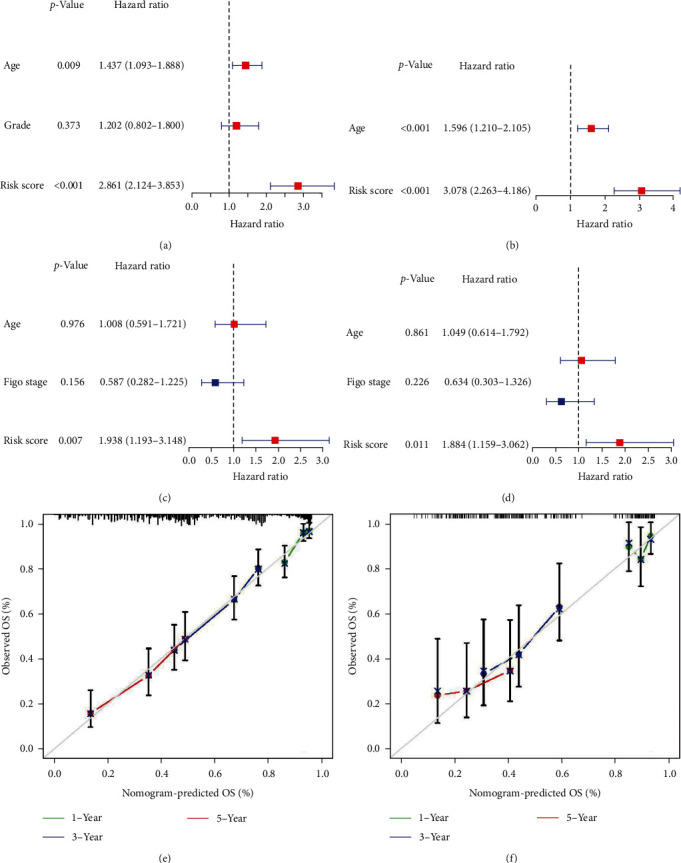
Joint analysis of risk scores and clinical characteristics. (a, b) Univariate (a) and multivariate cox regression analyses (b) for independent prognostic factors in TCGA. (c, d) Univariate (c) and multivariate cox regression analyses (d) for independent prognostic factors in ICGC. (e, f) The calibration curves of the nomogram in the training (e) and the testing sets (f).

Taking age, risk score, and grade into univariate and multivariate analyses using Cox regression, we recognized age and risk score as independent factors for OS in TCGA cohort (Figures 3(a) and 3(b)). In the ICGC cohort, although age showed little significance for prognosis, the RNA modification-related signature still displayed superior value for survival prediction in both univariate (hazard ratio (HR) : 1.938; 95% confidence interval (CI) : 1.193 − 3.148) and multivariate (HR : 1.884; 95% CI : 1.159 − 3.062) Cox regression analyses (Figures 3(c) and 3(d)). Furthermore, aware that a single risk score would contribute to the insufficient prediction; we devised a nomogram plot composed of risk scores and age. In the training cohort, there was a high degree of agreement between the observed and nomogram-predicted survival rates at 1, 3, and 5-year (Figure 3(e)). Similarly, the calibration curves of the nomogram showed good congruence between the *x*- and *y*-axes in the validation cohort (Figure 3(f)).

### 3.5. Molecular Mechanisms of the Difference between Two Risk Status

**Figure 4:**
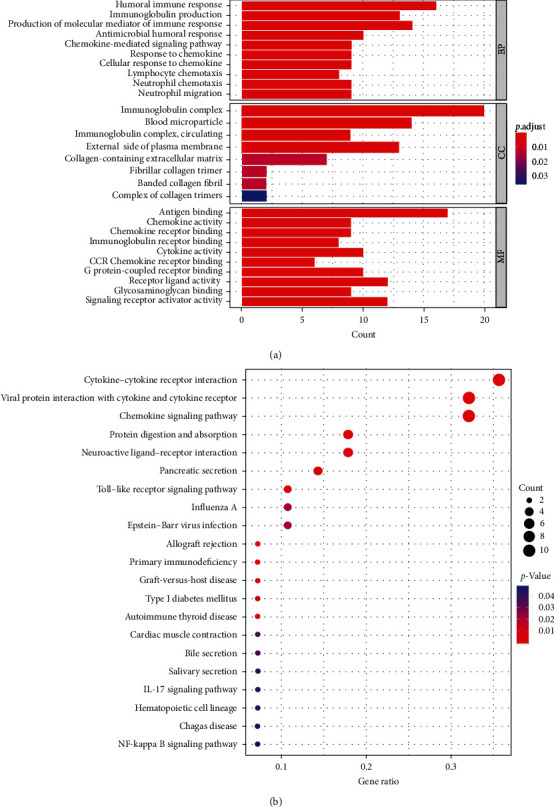
Functional analyses results. (a) GO enrichment analysis. (b) KEGG pathway enrichment analysis.

A total of 82 DEGs were used for GO and KEGG analyses (*Supplementary 4*). As shown in Figure 4(a), they are of paramount importance for immunological biological processes, such as “humoral immune response,” “immunoglobulin production,” and “production of molecular mediators of immune response”. And the cellular compounds results indicated that these genes participated in the formation of “immunoglobulin complex,” “blood microparticle,” and “immunoglobulin complex, circulating”. While “antigen binding,” “receptor ligand activity,” and “signaling receptor activator activity” were significantly enriched for the category of molecular functions. Essentially, KEGG results emphasized the connection between the signature and immune-related signaling pathways, including the IL-17 signaling pathway, chemokine signaling pathway, toll-like receptor signaling pathway, and NF-*κ*B signaling pathway (Figure 4(b)).

### 3.6. The Landscape of Immune Infiltration in Two OC Subgroups

**Figure 5:**
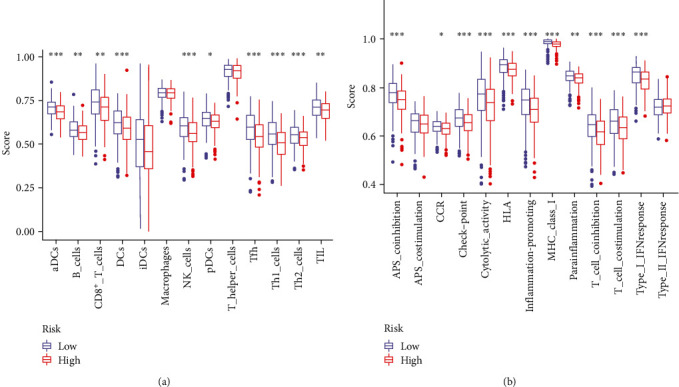
Comprehensive evaluation of immune profiles between low- and high-risk groups. (a) The qualification of immune cells infiltration. (b) The levels of immunological functions activities. The red color indicates high-risk samples, while the blue color indicates low-risk ones;  ^*∗*^*p* < 0.05;  ^*∗∗*^*p* < 0.01; and  ^*∗∗∗*^*p* < 0.001.

Considering that the risk score was close to immune signatures, we compared the immune diversity between the two risk states (Figure 5). Patients with high-risk scores tended to have reduced levels of tumor-infiltrating immune cells, involving dendritic cells (DCs), activated DCs, plasmacytoid DCs, CD8^+^ T cells, tumor-infiltrating lymphocytes (TILs), type 1/2 T helper (Th1/2) cells, T follicular helper (Tfh) cells, B cells, and NK cells (Figure 5(a)). In other words, these high-abundance immune cells might account for the favorable prognosis in OC patients. Moreover, all immune functions, which were significantly different between the two risk subtypes, exhibited more active patterns in low-risk patients, as expected (Figure 5(b)). In any case, the ssGSEA results demonstrated that low-risk scores were associated with enhanced immune profiles.

### 3.7. Chemotherapy Response Identification

**Figure 6:**
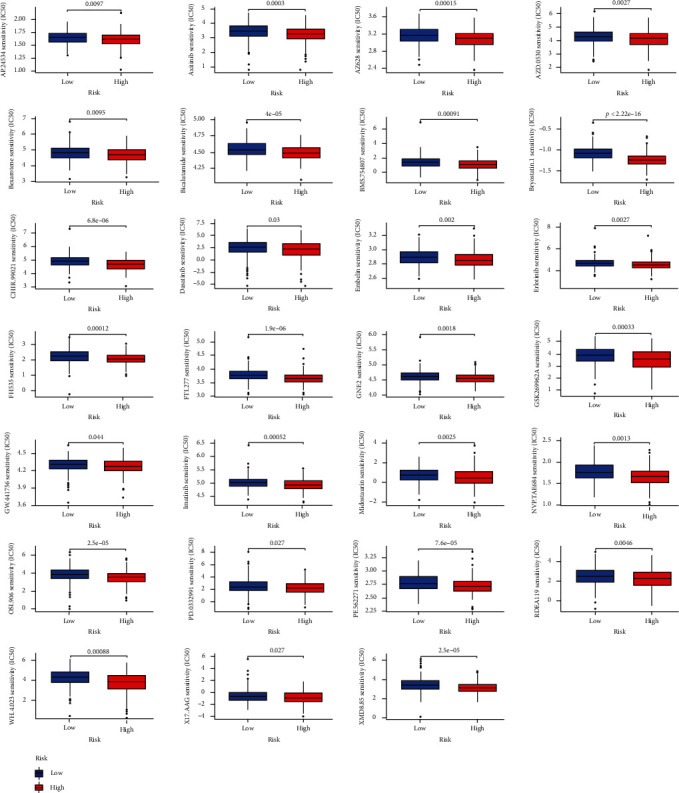
Medicaments with IC50 values significantly lower in high-risk individuals.

To further explore antitumor drugs, we collated IC_50_ values of several compounds based on the Genomics of Drug Sensitivity in Cancer database (https://www.cancerrxgene.org/). As a consequence, we found multiple medicaments more effective in high-risk people (Figure 6), which included ponatinib, axitinib, AZ628 (a pan-Raf kinase inhibitor), saracatinib, bexarotene, bicalutamide, BMS-754807 (an insulin-like growth factor-1R/IR inhibitor), bryostatin 1, CHIR-99021 (a potent GSK-3 inhibitor), dasatinib, embelin, erlotinib, FH535 (a PPAR and Wnt/*β*-catenin inhibitor), FTI-277 (an inhibitor of Farnesyltransferase), GNF-2 (a BCR-ABL inhibitor), GSK269962A (an inhibitor of ROCK), GW-441756 (a TrkA inhibitor), imatinib, midostaurin, NVP-TAE684 (an ALK inhibitor), linsitinib, palbociclib, PF-562271 (an inhibitor of FAK), refametinib, WH-4-023 (a dual LCK/SRC inhibitor), tanespimycin, XMD8.85 (an inhibitor of ERK5 and LRRK2). Admittedly, these discoveries extended future prospects of OC therapy, in which the needs of prolonging survival of high-risk patients may be satisfied.

## 4. Discussion

Over the years, m5C RNA modification has gradually emerged as a prominent channel that influences tumor initiation and progression. Simultaneously, efforts have been made to improve the feasibility of m5C RNA modification in predicting the prognosis of clear cell renal cell carcinoma [21], lung squamous cell carcinoma [15], triple-negative breast cancer [8], and prostate adenocarcinoma [22]. However, few studies have focused on its role as an exclusive biomarker for OC on the one hand [23]. However, there are also limitations to access m5C RNA modification-related genes, which are confined to writers, erasers, and readers [23]. In comparison, we performed methylated-RNA immunoprecipitation sequencing (MeRIP-seq) to identify related genes in our research, other than, as in the similar studies, the involved genes were directly obtained from the previous findings. Therefore, we have provided more reliable and comprehensive strategies. We attached great importance to m5C RNA modification, which has not yet been reported. In the current study, we identified differentially expressed m5C RNA modification-related genes between human OC and adjacent non-tumor tissues, which was devised for the first time using the MeRIP-seq method. By making full use of TCGA and experimental data, we established a prognostic model encompassing six novel RNA m5C-related genes: GALNT10, ATP1A3, PLA3G2D, GRIN2D, LAMP3, and GALNT6. Subsequently, we categorized the OC patients into low- and high-risk subtypes in terms of coef values and the corresponding gene expression levels in TCGA. Subsequent analyses reached an absolute consensus on the significantly negative correlation between risk scores and survival advantage, which was validated using the ICGC dataset. In addition, the immune profile was dramatically different between the low- and high-risk populations. In conclusion, our results not only lay a solid foundation for the refinement of prognostic stratification but also exert positive effects on the immunotherapy in OC patients.

GALNT10 belongs to the N-acetylgalactosaminyltransferase (GalNAc-T) family, the encoded protein of which plays a catalytic role in O-glycan synthesis [24]. Subtle alterations in GALNT10 give rise to aberrant O-glycosylation, thus facilitating tumor cell proliferation in hepatocellular carcinoma and gastric cancer [24, 25]. In vitro cell assays have shown that the stem cell-like characteristics of OC cells are regulated by GALNT10, hinging on its glycosyltransferase property [26]. In addition, it has been reported that OC patients are more likely to experience dismal prognosis and immune suppression with elevated GALNT10 expression [27]. Moreover, it dovetails precisely with our findings. Regrettably, the role of GALNT10 in the regulation of RNA modification remains unclear. However, it must be noted that GALNT10 represents an adverse factor for OC patients' survival, according to our data. Similarly, it is commonly recognized that GALNT6, another GalNAc-T family member, impedes tumor growth and progression when losing expression [28, 29]. Interestingly, previous studies have emphasized that OC patients tend to experience adverse outcomes upon increased GALNT6 expression [30, 31], which is in contrast to our present report. Undoubtedly, a completely different argument does cause a controversial issue about the GALNT6 functions of in OC prognosis, awaiting further exploration. As a widespread transmembrane protein, Na^+^/K^+^-ATPase (NKA) is responsible for the maintenance of the electrochemical gradient across plasma membranes, of which ATP1A3 encodes the *α*3 subunit [32]. It is commonly believed that pathogenic ATP1A3 variants have resulted in a variety of neurological disorders in the last 20 years, such as rapid-onset dystonia parkinsonism, alternating hemiplegia of childhood, and cerebellar ataxia [32]. By targeting ATP1A3, Y-box binding protein 1 (YB-1), and RNA-binding protein Human Antigen R (HuR) participate in the formation of RNA-dependent complexes on m5C modified nucleotides [33]. In addition, recent studies have confirmed that ATP1A3 activation not only enhances temozolomide sensitivity but also inhibits the proliferation of glioma cells immediately [34, 35]. In contrast, overexpressed ATP1A3, which promotes tumor invasion, has been identified in clinical specimens of gastric cancer [36]. More importantly, a recent study indicated that OC patients were more likely to suffer from poor OS when ATP1A3 was upregulated, which agrees well with our findings [37]. Phospholipase A2 (PLA2) proteins are a group of lipid metabolism-related molecules with catalytic properties of that hydrolyze membrane glycerol phospholipids and are therefore essential for various biochemical processes, such as the response to inflammation [38]. On the one hand, PLA2G2D is responsible for encoding a member of secreted PLA2, group IID PLA2, which is associated with resistance to immune checkpoint blockade as well as limitation of the immune system in primary solid tumors [39, 40]. In contrast, a positive correlation between immune infiltration and PLA2G2D expression has been identified in cervical cancer [38]. In addition, upregulation of PLA2G2D improves the prognosis of cutaneous melanoma [41], breast cancer [42], and head and neck squamous cell carcinoma [43]. Together with our results, PLA2G2D represents a good chance of acting as a beneficial regulator rather than an immunosuppressive role in OC. Encoding a subunit of the N-methyl D-aspartate receptor, GRIN2D is highly expressed in hepatocellular cancer and can exert an obstacle effect on the immune response, thus promoting tumor growth [44]. Additionally, GRIN2D can be used as an executor controlled by miR-129-1-3p to regulate breast cancer cell infiltration and migration [45]. Herein, elevated GRIN2D expression was dramatically detected in neoplastic tissues along with a potential marker for poor therapeutic effects in OC. LAMP3, a lysosome-associated membrane protein, is indispensable for sustaining the integrity of lysosome structure and related functions [46]. A recent study indicated that excessive LAMP3 activation could induce the degradation of other transmembrane proteins of the lysosome, which in turn increases membrane permeability, leading to caspase activation and autophagy inhibition [47]. More importantly, LAMP‑3(+) DCs stimulate the proliferation of tumor infiltrating cells CD8 which symbolizes a favorable prognosis in esophageal squamous cell carcinoma [48]. Conversely, they also cause tumor aggressiveness and resistance to endocrine therapy in breast cancer, indicating the opposite effects on cancer types [49]. Although the specific mechanism of LAPM3 in OC cells remains unclear, we preliminarily conclude that LAMP3 serves as a beneficial gene for prolonging the survival of OC patients.

Recently, m5C RNA modification has been recognized to play a role in immune cell biological processes [50]. First, GO analysis showed that the DEGs between the two risk groups objectively participated in the process of canonical immune responses and the formation of prominent immunoglobulins, which also have an impact on antigen and cytokine binding. The signaling pathways, “Chemokine signaling pathway,” “IL-17 signaling pathway,” “Toll-like receptor signaling pathway,” and “NF-kappa B signaling pathway” are all associated with immunomodulation and tumor metabolism. To some extent, functional annotation suggests that immune status can be essentially distinguished between the two risk groups in an m5C RNA modification manner.

In this study, we also noticed that immune cells and related functions were widely retarded in the high-risk group displayed on GSEA results, implying that antitumor immunity is perturbed in OC patients with high-risk scores. As specialized antigen-presenting cells, mature DCs, when stimulated by antigens, activate CD8^+^ T cells to function [51]. The density of CD8^+^ T cells is well known as the most essential antitumor effector associated with superior survival in OC patients [52]. It should be noted that type I IFN secreted by pDCs is a vital anti-cancer cytokine that activates and recruits NK and B cells [53, 54], although pDCs are generally considered intruders in the regulation of T cell responses to cancers [55]. In addition, Tfh cells have been positively correlated with prognosis owing to the promotion of B cell maturation and maintenance of CD8 T cell-dependent antitumor activity. The characteristics of Tfh cells in OC patients remain further refined, but we found that the group with prolonged survival appeared to carry more conspicuous Tfh cell infiltration in OC. Th 1 cells, one of the Th cell subsets, participate in antitumor immunity through the production of inflammatory mediators to eradicate cancer cells directly, while simultaneously promoting the activation and recruitment of NK cells [56, 57]. Th1 cells are particularly relevant for extending recurrence-free survival in OC patients [58]. In addition, a recent update revealed a distinct effector mechanism in that Th 2 cells induce terminal differentiation to osteoblasts and initiate breast cancer [59].

Over the past several years, blocking immune checkpoints has reframed the treatment of a variety of malignancies and has led to remarkable improvements in OS [60]. Accordingly, it seems to be more efficient for the low-risk population to benefit from immune checkpoint inhibitors, as they harbor a greater density of immune checkpoints that mediate immune escape. The immune escape mechanism of multiple solid tumors is mainly due to the loss of antigenicity induced by the deficiency of major histocompatibility complex class I (MHC I)/human leukocyte antigen (HLA) [61–63]. In contrast, an increase in tumor-specific antigen presentation recognized by T cells on HLA/MHC I molecules has been deemed to improve survival outcomes [62]. Our signature revealed impaired HLA/MHC I functions in high-risk OC patients. Moreover, MHC I can hardly be upregulated in poorly differentiated tumor cells under corresponding conditions for activation, a signature that is likely unique to OC [63]. Hence, there is presumably stratification of differentiation between our risk groups to some extent. Alongside these, the higher portion of antitumor-associated immune responses has been observed in the low-risk cohort, such as “APC co inhibition,” “cytolytic activity,” “CCR,” “parainflammation,” “flammation-promoting,” “T cell co-stimulation/inhibition,” and “Type I IFN Reponse.” Overall, the immune profile revealed bridges the gap between immunotherapy and m5C RNA modification, which is of clinical significance in strengthening the applications of immunotherapy in OC.

In addition, we have identified more effective chemotherapeutic drugs for high-risk patients, which may lay a solid foundation for prolonging the survival of the overall population with OC.

## 5. Conclusion

In conclusion, we have described the survival prediction patterns of six m5C RNA methylation-related genes in OC derived from MeRIP-seq. Based on TCGA, our signature could predict the survival profiles and immune infiltration status of OC patients independently, which has been significantly reproduced in a separate database. More importantly, our findings are expected to provide a novel horizon for stratified management and brilliant immunotherapeutic strategies as well as potential adjuvant chemotherapy for high-risk patients. However, the detailed regulatory mechanism of the candidate genes in m5C RNA methylation remains unclear, and further experimental studies are required.

## Figures and Tables

**Table 1:** The risk coefficient value of each core gene.

Gene	Coef
ATP1A3	0.130955704
GRIN2D	0.236142949
PLA2G2D	−0.373104088
GALNT10	0.448763921
LAMP3	−0.162535052
GALNT6	−0.069757800

*Note*. Coef, coefficient.

## Data Availability

The data supporting the findings of this study are openly available online. Transcriptome profiling and clinical data of OC patients were downloaded from TCGA (https://portal.gdc.cancer.gov/), the Genotype-Tissue Expression (GTEx) database (https://xenabrowser.net/), and ICGC (https://dcc.icgc.org/). In total, 88 normal ovary tissues in the GTEx, 379 OC samples in the TCGA-OV cohort, and 93 samples in the ICGC-OC-AU cohort were used for further analysis.

## Supplementary Materials

Supplementary 1 m5C RNA modification-related genes derived from the transcriptomic and methylome data.

Supplementary 2 The forest plot of m5C RNA modification-related genes correlated with survival time.

Supplementary 3 The slightly positive correlation between PLA2G2D and m5C RNA modification regulators.

Supplementary 4 The DEGs were used for functional enrichment analysis.
