# Supplementary material for: Contribution of m5C RNA Modification-Related Genes to Prognosis and Immunotherapy Prediction in Patients with Ovarian Cancer
Source: Mediators Inflamm. 2023 Nov 13;2023:1400267. doi: 10.1155/2023/1400267 (PMC10661868; doi:10.1155/2023/1400267)
Supplement: Supplementary 1 — m5C RNA modification-related genes derived from the transcriptomic and methylome data. [file 1400267.f1.docx]

Table S1. m5C RNA modification-related genes derived from transcriptomics and methylome data.

| Gene |
| --- |
| MMS19 |
| ST8SIA5 |
| ANKS1A |
| CLPB |
| HPSE2 |
| FEZF1 |
| PLCE1 |
| ATOX1 |
| RYR3 |
| MLC1 |
| DLG1 |
| ZFP92 |
| MT1B |
| SMARCD3 |
| C10orf90 |
| ZHX3 |
| PRCP |
| FAM153A |
| GRAP2 |
| IFT52 |
| ZNF684 |
| KLHL13 |
| TESMIN |
| OR1L6 |
| COX7A2L |
| MYO1B |
| ABCC6 |
| ZPLD1 |
| PRTG |
| IRS4 |
| KLHL2 |
| NAGPA |
| ADAMTS18 |
| SEPT14 |
| SLC52A1 |
| NUP155 |
| MATK |
| CHRDL1 |
| PRDM14 |
| SIDT1 |
| SUSD5 |
| EPB41L4B |
| APOBEC3D |
| GNAL |
| MATR3 |
| KIF7 |
| LYG1 |
| ZAR1L |
| SERPINB5 |
| LGR6 |
| RTP3 |
| NLRC4 |
| FAM234B |
| SLC15A2 |
| NMS |
| MS4A10 |
| IFT46 |
| MAP3K3 |
| KCTD15 |
| NUDT17 |
| UNC5A |
| SERPINA12 |
| FER1L5 |
| IGSF3 |
| SLC13A1 |
| MYO3B |
| PROM2 |
| DUSP7 |
| C6orf223 |
| HOXA13 |
| NANOG |
| ZNF347 |
| FKBP7 |
| TCEANC2 |
| STRADB |
| DMC1 |
| PCYT2 |
| HES5 |
| GPNMB |
| MAB21L3 |
| FGFBP3 |
| DAD1 |
| KIAA1210 |
| NSD2 |
| PSG8 |
| KCNT1 |
| PTPN22 |
| NXPH3 |
| UNC45B |
| OR14A16 |
| ZNF506 |
| ERICH6B |
| TUBAL3 |
| TGM2 |
| ZYG11A |
| ITM2B |
| PRKCB |
| STK26 |
| ACTL6A |
| CYP2C19 |
| NOS1 |
| ARPP21 |
| PALLD |
| SPATA45 |
| OR4D9 |
| CSNKA2IP |
| CPLX4 |
| TRPM6 |
| TRIM43B |
| CD160 |
| SEC14L5 |
| FEV |
| CCDC153 |
| LRRC8C |
| RPGRIP1 |
| PRAMEF13 |
| ALDOC |
| PRDM2 |
| NUP93 |
| KCNA1 |
| CD1A |
| SCGB1C1 |
| SRRM4 |
| TDRD15 |
| CFAP46 |
| FBXO22 |
| CST7 |
| SLC2A13 |
| OR8J3 |
| SHROOM1 |
| DAP3 |
| TBXT |
| ADGRE3 |
| ATP8B2 |
| MDH1 |
| STAU2 |
| ATP6V0B |
| DPH3 |
| SKP1 |
| CD38 |
| PGRMC2 |
| TNFRSF9 |
| UBE2I |
| PAQR6 |
| TMEM63C |
| PGBD5 |
| AVPR1B |
| MUC3A |
| CTIF |
| ITGB1BP2 |
| ISM1 |
| SPRR2D |
| TM4SF4 |
| SLC16A12 |
| PRAMEF15 |
| USP14 |
| ALK |
| ZC2HC1B |
| RBM24 |
| MSH6 |
| RASGRF1 |
| TNNT3 |
| GON4L |
| SLC6A15 |
| BTBD17 |
| MAPK12 |
| UBA7 |
| FRMD5 |
| LMBRD1 |
| IQCM |
| BTRC |
| TRIM2 |
| SHBG |
| PAK6 |
| SEMA3E |
| MXD4 |
| SPRYD4 |
| DNASE1L3 |
| DNAH6 |
| CHMP1B |
| MAP3K13 |
| RBFOX1 |
| ALKAL1 |
| TRIM72 |
| PCBP4 |
| CAMTA1 |
| PRAMEF26 |
| ARID3A |
| MYO15B |
| ORC1 |
| TNFRSF11B |
| LRRC4C |
| C3orf56 |
| MALRD1 |
| KIAA1324 |
| AIPL1 |
| TMCO5A |
| PNMA5 |
| SNX19 |
| SEC14L1 |
| TRIM29 |
| DUSP5 |
| MPP4 |
| TNFSF15 |
| TIMM22 |
| ZNF41 |
| LIMD1 |
| C14orf132 |
| TF |
| TDRD5 |
| CAPN14 |
| GCK |
| FBXO24 |
| CELA1 |
| TNNC2 |
| DNAH12 |
| DAO |
| SLC9C1 |
| ANHX |
| PDCL2 |
| TMEM202 |
| ODF3L2 |
| ADAM23 |
| CXCR2 |
| NBPF19 |
| LIPE |
| RNASE2 |
| AP000790.2 |
| ADCY5 |
| TEX261 |
| ESYT3 |
| HVCN1 |
| AL845331.2 |
| FAT2 |
| DDHD2 |
| CTSV |
| PRRC2B |
| OR10K1 |
| RYR1 |
| PCDH18 |
| SLAMF1 |
| DPYD |
| UNC13B |
| OR13C4 |
| PRMT2 |
| ATAD3B |
| SCN4A |
| GOLGA6D |
| LAMB4 |
| FAM24A |
| EPHA5 |
| FLNC |
| TMCC2 |
| PACS2 |
| IZUMO2 |
| HAL |
| CATSPERG |
| SIPA1L2 |
| ATP7A |
| RPL32 |
| HOXB13 |
| CST3 |
| COL7A1 |
| COLGALT2 |
| FCGBP |
| ATP5PD |
| TBXA2R |
| AKNA |
| PKHD1 |
| PAPPA |
| OR8A1 |
| SLC10A4 |
| SLC7A14 |
| RAB26 |
| AWAT1 |
| NEBL |
| PNMA8C |
| ZWILCH |
| TP73 |
| KRTAP17-1 |
| NQO2 |
| TRPC7 |
| KCNQ5 |
| ENAM |
| MPV17L |
| ATP13A4 |
| ZNF639 |
| CACNG4 |
| AKR1C2 |
| GFI1B |
| IPO13 |
| MYH13 |
| MAPT |
| ENAH |
| HSPA4L |
| GABRQ |
| PLXNC1 |
| NR2C2 |
| OSBP |
| SYNE4 |
| TMEM230 |
| ALDH1A2 |
| PCSK6 |
| KIAA1755 |
| PABPC3 |
| ADRA1A |
| TEX13D |
| C19orf67 |
| AKR7A3 |
| EPB41L1 |
| KIAA0586 |
| RNASE10 |
| SNX31 |
| IL5RA |
| TIAM1 |
| TEKT3 |
| SCN11A |
| CDH24 |
| KIAA1549L |
| PHOX2B |
| AC136428.1 |
| ZNF343 |
| ATP10A |
| MFRP |
| CC2D1A |
| PHACTR1 |
| ELOVL7 |
| TTC21B |
| PCLO |
| SAMD7 |
| SORCS2 |
| OPA3 |
| C11orf53 |
| ZNF595 |
| ACTR6 |
| TMEM171 |
| AGO4 |
| MILR1 |
| HUS1 |
| KIF19 |
| GALR2 |
| FNDC7 |
| PPP1R1B |
| SV2B |
| KCNB2 |
| ADGRL1 |
| HECW2 |
| FAM149A |
| LSM3 |
| MOGS |
| PKD1L2 |
| USP13 |
| PPM1M |
| TBX21 |
| RRAD |
| FAM110A |
| TNFAIP6 |
| SLC19A3 |
| PGLYRP2 |
| CFAP58 |
| TENM1 |
| CRYBA2 |
| RALGPS1 |
| NCOA3 |
| SNAI1 |
| NBPF20 |
| TMEM212 |
| ZAN |
| STEAP4 |
| EYA1 |
| SERPINB2 |
| TXNRD2 |
| HOXB5 |
| ARGFX |
| S100A3 |
| WDR17 |
| OR6C74 |
| ZFHX4 |
| C22orf42 |
| MUC5B |
| MUC12 |
| DCTN1 |
| SVEP1 |
| CAMK2B |
| TLL2 |
| CDH18 |
| RP1 |
| GRIP1 |
| SUB1 |
| IGSF9 |
| STC1 |
| DEFB124 |
| AKTIP |
| IKBKG |
| SPOCK3 |
| ARL10 |
| KIR3DX1 |
| GLDN |
| JPH2 |
| NEDD4L |
| ZBTB45 |
| ST8SIA1 |
| SLC2A10 |
| EGF |
| CPSF3 |
| ATP2B2 |
| APOA5 |
| IL1A |
| CACNA1H |
| CNRIP1 |
| MYH3 |
| CFC1B |
| MYO1H |
| KCND3 |
| HARS |
| NALCN |
| TEX33 |
| SH3GL3 |
| KLHL21 |
| RIMS3 |
| ITGB1 |
| ASTN2 |
| EXOC3L2 |
| RPN1 |
| NUGGC |
| KCNH4 |
| DNAJC16 |
| SIX2 |
| CSPP1 |
| RAB9B |
| PCDHGC4 |
| NCAPG2 |
| CST9L |
| DCT |
| KLHL28 |
| OVCH2 |
| ACRBP |
| BEST1 |
| PTK7 |
| HSD3B1 |
| ATG5 |
| GAB4 |
| LMAN2L |
| SLC50A1 |
| TACR2 |
| CD207 |
| PPOX |
| CLEC2A |
| EDDM3B |
| SERTAD1 |
| TM9SF4 |
| HSPA6 |
| ROPN1 |
| CHAT |
| NT5DC4 |
| TTBK1 |
| NKIRAS1 |
| KYNU |
| SCGN |
| AKAP2 |
| SVOP |
| STK32A |
| KRTAP10-6 |
| LRP1B |
| AK9 |
| MYH10 |
| ZNF578 |
| FBN3 |
| SDR16C5 |
| ABCG8 |
| CTBP1 |
| ZNF442 |
| MYLK4 |
| CASC10 |
| OR10J1 |
| PLAC9 |
| OTUD7A |
| AMPD1 |
| KALRN |
| FOXR1 |
| MATN3 |
| OR8K3 |
| ATP6V0A2 |
| GAGE12G |
| DOPEY2 |
| OR6J1 |
| RAB11FIP4 |
| C1QTNF7 |
| PSENEN |
| OPN3 |
| FAM196B |
| NRAP |
| SLC25A34 |
| EGFLAM |
| INSM2 |
| HBD |
| C17orf80 |
| CFC1 |
| TCF19 |
| SULT2A1 |
| TMEM214 |
| MRPS22 |
| GNG4 |
| NAV2 |
| PLD5 |
| TMC1 |
| PAK3 |
| EFCAB5 |
| STK3 |
| TCFL5 |
| WASHC1 |
| TGFBR1 |
| GARNL3 |
| LRRC34 |
| RNF150 |
| CLPS |
| ZNF524 |
| SFMBT2 |
| COL19A1 |
| IL18 |
| TMEM169 |
| CYP2A7 |
| NKAP |
| TIMM29 |
| ABI3BP |
| TOGARAM2 |
| NEURL3 |
| EXOSC3 |
| ANKRD54 |
| C19orf81 |
| CLDN10 |
| GCNT2 |
| HPCAL4 |
| SLCO1A2 |
| NACA |
| SUGP1 |
| DAPK2 |
| AXDND1 |
| LRRC15 |
| CNFN |
| CALB2 |
| HAPLN2 |
| FLI1 |
| SEL1L2 |
| TNFRSF19 |
| OR52A1 |
| THEGL |
| MAP7D2 |
| PLCXD3 |
| TRIM69 |
| ZNF479 |
| JPH4 |
| IGFN1 |
| LUZP2 |
| ECHDC3 |
| OR4C45 |
| OBSCN |
| CPA4 |
| CLCA2 |
| FAM155A |
| KCNAB1 |
| ANKRD40 |
| HHIPL2 |
| C10orf95 |
| ITK |
| EIF3F |
| ZNF514 |
| TRIB1 |
| ADGRE2 |
| ESRRB |
| FAM153C |
| GAP43 |
| ARSK |
| GNG2 |
| ARFGAP2 |
| CENPN |
| ZNF536 |
| OR6Y1 |
| RCAN3 |
| FTSJ3 |
| STYX |
| TAF4 |
| TEX14 |
| CPB1 |
| DRP2 |
| CSMD1 |
| G0S2 |
| MAP6 |
| PDZD7 |
| RETNLB |
| NYAP1 |
| PLEKHH2 |
| WDR64 |
| PARVB |
| DRGX |
| SLC24A2 |
| MFAP3L |
| ADCY8 |
| SCARB1 |
| ARHGAP19 |
| ZBTB7C |
| MSANTD1 |
| MME |
| PHF24 |
| SENP3 |
| ZIC3 |
| KANK4 |
| GJB7 |
| CEP72 |
| SGO1 |
| PCDHB13 |
| KRT33B |
| PGLYRP4 |
| OSBPL6 |
| FZD10 |
| FRMD4A |
| LCMT2 |
| ORC5 |
| MEIS3 |
| BCL11B |
| NTRK3 |
| KIAA1841 |
| AGBL1 |
| ACAA2 |
| WDR38 |
| ABLIM2 |
| FCMR |
| ZNF444 |
| LARP6 |
| RXFP1 |
| DCAF8L1 |
| RAB7B |
| FBN1 |
| PLOD3 |
| HAVCR1 |
| ABCA12 |
| AC005324.3 |
| GP2 |
| TPST2 |
| SIRPD |
| NXPE2 |
| MRC1 |
| NUTM2F |
| TMEM178A |
| SLC6A14 |
| GPR160 |
| ZFP82 |
| COCH |
| SLC14A2 |
| CTNNA3 |
| KLK9 |
| HSDL1 |
| ICAM2 |
| ASAH2B |
| SPATS2 |
| NOMO3 |
| GNGT1 |
| MBLAC1 |
| ARNTL |
| ALAS1 |
| NAA40 |
| KIF5C |
| ADAMTS17 |
| NDUFV2 |
| ZNF696 |
| CNKSR2 |
| BEND4 |
| NEK10 |
| AMPD3 |
| FAF2 |
| ADCYAP1R1 |
| SNX29 |
| TSEN54 |
| AGAP2 |
| CDH20 |
| CFAP20 |
| CA12 |
| NEB |
| ABI3 |
| COL13A1 |
| ALDH5A1 |
| EIF5AL1 |
| MS4A6E |
| C8orf59 |
| EPHX2 |
| CORO6 |
| STYXL1 |
| IL18RAP |
| STMN1 |
| CDC7 |
| GABRA1 |
| PIFO |
| HTR3D |
| REELD1 |
| RPH3A |
| LSAMP |
| STK39 |
| FOXK1 |
| TRPC5OS |
| CFAP74 |
| GCDH |
| ADCY1 |
| NR2F2 |
| TMEM94 |
| ZMYM1 |
| GRID1 |
| SRL |
| INTS5 |
| HMBS |
| EFR3B |
| FAM186A |
| RNF165 |
| DNAH2 |
| ADAMTS12 |
| ZNF215 |
| ZNF287 |
| ZFR2 |
| FER1L6 |
| BIN1 |
| UBASH3A |
| OR2AG1 |
| PLXNA4 |
| NMRAL1 |
| DGKK |
| ACSS2 |
| GALNT15 |
| KIF23 |
| ACAP1 |
| ATL1 |
| PLIN1 |
| SH2D4A |
| ARMCX3 |
| SPINDOC |
| ZNF333 |
| AGO1 |
| SPINK2 |
| HLCS |
| ITGAM |
| ROCK1 |
| TBC1D24 |
| ZNF695 |
| BCO1 |
| NXPH4 |
| DCDC1 |
| IQGAP3 |
| SHC2 |
| MTUS2 |
| VASH2 |
| MGAM |
| IL19 |
| ZBTB42 |
| GOLGA8M |
| NDUFB10 |
| CCNO |
| RXRG |
| DNAH10OS |
| ASGR2 |
| PRKAA2 |
| ADARB1 |
| SGPP2 |
| KDR |
| DUSP19 |
| CGB7 |
| RGPD4 |
| SORCS1 |
| COL5A3 |
| ALDH1L1 |
| HAS3 |
| CCL23 |
| BPGM |
| HOXD8 |
| FAM207A |
| ZDHHC11 |
| KRT9 |
| FOXP2 |
| CNNM1 |
| GDPD4 |
| POLR3A |
| EXT1 |
| ADGB |
| ARHGEF37 |
| CDRT1 |
| DLG2 |
| PPIH |
| MMP3 |
| DSC3 |
| PDE2A |
| CRHR1 |
| ZIC5 |
| B3GNT3 |
| SAP130 |
| LAYN |
| CNR2 |
| SHE |
| DNAH7 |
| IFIT1B |
| MYH15 |
| ZNF365 |
| PIGH |
| TEX13C |
| DENND2C |
| PLCG2 |
| EXOSC1 |
| SLC22A17 |
| CDYL2 |
| LRRTM4 |
| SPATA31A3 |
| ZNF597 |
| MYOM2 |
| RANBP3L |
| VKORC1L1 |
| TUNAR |
| EXD1 |
| OR2V1 |
| TG |
| ZNF619 |
| SYCP2L |
| GLRA1 |
| SCPEP1 |
| FAAH2 |
| PKN3 |
| SFXN1 |
| TRPC5 |
| RFPL3 |
| ZNF428 |
| MYO3A |
| GCC1 |
| TIMM9 |
| IFNA17 |
| KIAA0513 |
| SH3BP2 |
| MYO1A |
| THEM5 |
| MYRF |
| ZNF705D |
| TSKU |
| PYGB |
| SNPH |
| EPN3 |
| B3GNT4 |
| TXNRD3NB |
| SENP2 |
| ANAPC2 |
| KCNS1 |
| CBLL2 |
| KCNJ4 |
| JAML |
| KIF6 |
| RFT1 |
| DGKI |
| NFIX |
| ITGAD |
| CALCB |
| KRT4 |
| NAE1 |
| UBL3 |
| TRIM4 |
| CDH16 |
| IQCE |
| TRIM49 |
| ICMT |
| ZSWIM2 |
| GJA3 |
| LINS1 |
| TAT |
| PACSIN1 |
| TBC1D30 |
| GALNT6 |
| TMPRSS5 |
| BTN2A2 |
| LIFR |
| TESPA1 |
| THBS4 |
| CACNG8 |
| CHST9 |
| SLC35E4 |
| METTL6 |
| SPNS2 |
| NCAM2 |
| B4GALNT3 |
| DEFB132 |
| MYO15A |
| SAMD14 |
| IL1R2 |
| DZIP1 |
| PIK3CA |
| MAGI2 |
| EXPH5 |
| ATP6V1E2 |
| MAG |
| FLT3 |
| NELL1 |
| GALK2 |
| AKAP6 |
| ANKRD30B |
| CEP97 |
| C16orf91 |
| MINDY4 |
| GCSAML |
| GRIK5 |
| TRIM64B |
| ERBB2 |
| CYP2C18 |
| LNPK |
| FLG2 |
| GRIN2D |
| FREM3 |
| GALNTL6 |
| FUT9 |
| SRGAP2B |
| OSBPL7 |
| FUT3 |
| PXDNL |
| AC115220.1 |
| DBI |
| TAAR5 |
| TDRD12 |
| LIMCH1 |
| COX18 |
| ASIC2 |
| SOHLH2 |
| NAT2 |
| SLITRK3 |
| EML6 |
| CHRDL2 |
| PRG2 |
| WDR87 |
| C1QTNF6 |
| LINGO2 |
| INTS9 |
| RTL9 |
| SPDEF |
| SFI1 |
| EDDM3A |
| NKD2 |
| MGAT4C |
| KIAA1257 |
| FKBP3 |
| ADGRG2 |
| IL36A |
| OR4D6 |
| ING4 |
| PTH2R |
| IFI30 |
| PLEKHM1 |
| AP1B1 |
| WNT10A |
| LIPG |
| CD163L1 |
| LAD1 |
| PLEKHG4B |
| NGLY1 |
| EPHX1 |
| LGI1 |
| GABRP |
| ADGRL3 |
| BORCS6 |
| TACSTD2 |
| SIL1 |
| PRTN3 |
| NR5A2 |
| OXGR1 |
| MICAL2 |
| ROBO4 |
| GOLGA8K |
| KLRC3 |
| CHRNB3 |
| PPP1R36 |
| PKD1L1 |
| SLC22A25 |
| NLGN1 |
| EGFL6 |
| DGAT2 |
| TSNARE1 |
| BCL2L1 |
| CEBPG |
| COL6A5 |
| DNAH9 |
| GTF3C4 |
| FAM69A |
| PIGN |
| JARID2 |
| OR11H1 |
| HEATR9 |
| SHISA9 |
| DBF4B |
| KIF27 |
| EBF2 |
| SPATA18 |
| SSX1 |
| CDC45 |
| SMPD3 |
| OR4K17 |
| CALN1 |
| NEXN |
| HLA-G |
| RGS13 |
| SMIM36 |
| LRP2 |
| SPTA1 |
| UNC13A |
| ANK1 |
| CCDC136 |
| SLC24A4 |
| EDC4 |
| FOXI3 |
| ZCCHC17 |
| TNN |
| MPZL3 |
| GOLGA6L6 |
| IL22RA1 |
| POLR1D |
| PCDH8 |
| LRRC37A3 |
| DPP9 |
| TNIP2 |
| NOL6 |
| DMRT2 |
| EXD3 |
| FAH |
| SFTPB |
| CARTPT |
| DNM2 |
| GLDC |
| EIF3C |
| CNTNAP2 |
| TBX19 |
| PLA2G7 |
| KIF1C |
| CDK5R1 |
| GABRR1 |
| MCM2 |
| FUCA1 |
| RYR2 |
| FAM205C |
| FAM72A |
| ACAD10 |
| OR2A25 |
| CCDC169 |
| ATP2B4 |
| KIAA0895 |
| GALNT14 |
| TMEM170B |
| ROBO3 |
| PRLR |
| TMPRSS11F |
| CPLX2 |
| PANX2 |
| WFDC11 |
| CYP11B1 |
| MBD3L5 |
| CSPG5 |
| TBX20 |
| MTO1 |
| ASB18 |
| RIPOR3 |
| NSMCE1 |
| FAT3 |
| POP1 |
| NPSR1 |
| CACNB2 |
| STXBP4 |
| MAP3K4 |
| MS4A18 |
| SRC |
| PRAMEF25 |
| HHLA1 |
| PET100 |
| MLF2 |
| GOLGA6L4 |
| DSG3 |
| AL365214.3 |
| ARL3 |
| SLC12A5 |
| SRPX |
| NR1H4 |
| MAPRE2 |
| PKD1L3 |
| SEC14L2 |
| PHEX |
| RFX6 |
| IQCF3 |
| PTPRO |
| DNAJB2 |
| TRIM49D2 |
| OTOGL |
| TFPI |
| CXorf36 |
| ISX |
| ABCA4 |
| POTEM |
| KLHDC10 |
| MAT1A |
| GFAP |
| TMEM108 |
| PDE4DIP |
| LGI3 |
| ZDHHC14 |
| RSPH10B |
| CYP26B1 |
| LRRC1 |
| ZFP64 |
| SCG3 |
| ZSWIM5 |
| ASIC4 |
| CYB5R2 |
| WFIKKN2 |
| OR4C6 |
| TSPAN18 |
| SAMD15 |
| GABRB3 |
| CCDC61 |
| GOSR2 |
| MSTN |
| AZIN2 |
| ASB14 |
| TACR3 |
| SLC35C1 |
| MB21D2 |
| DNASE1L2 |
| HFE |
| MOGAT1 |
| SYDE1 |
| GRM7 |
| MED12L |
| AC008687.4 |
| MKI67 |
| DNAH8 |
| ADAP2 |
| CNTNAP5 |
| SPP2 |
| AL772284.2 |
| EDARADD |
| CLSPN |
| ACTG2 |
| CDH7 |
| RPL35A |
| OR10H3 |
| RAB40C |
| ACOXL |
| LRCH1 |
| LLPH |
| TTN |
| AMN |
| PDK1 |
| PGPEP1L |
| ITGA11 |
| BUD13 |
| UNC80 |
| AC135068.1 |
| PPP1R16B |
| KCNV1 |
| IQSEC3 |
| PNLIPRP3 |
| HHIP |
| ILDR2 |
| DYRK1A |
| EFCC1 |
| SCAF1 |
| TRAF3IP3 |
| DNAH3 |
| ST3GAL3 |
| HFE2 |
| PRKCQ |
| CHD5 |
| TIRAP |
| SLC6A12 |
| FAM214B |
| OR51A4 |
| EVA1A |
| GABRG2 |
| FCER2 |
| F5 |
| ATP12A |
| ISLR2 |
| ITGA9 |
| MEI1 |
| KRTAP4-7 |
| ULK2 |
| PIAS4 |
| LYRM4 |
| UGT2B11 |
| EXO1 |
| PATE3 |
| CUL4A |
| ENPP6 |
| FSTL3 |
| QSOX1 |
| PIK3R1 |
| LRRC74A |
| CDKN2C |
| PDGFRA |
| PKDCC |
| CFAP65 |
| MFSD13A |
| CES5A |
| OVCH1 |
| ARHGAP36 |
| LYSMD1 |
| SEPT1 |
| PDLIM3 |
| CRYL1 |
| OTC |
| RPP25L |
| SCN8A |
| ABCB11 |
| NMT1 |
| KCNC2 |
| IDH1 |
| LDLRAD1 |
| MUC21 |
| CDCP1 |
| PITX3 |
| TIMM50 |
| PGM5 |
| EYA4 |
| COL26A1 |
| ANKRD20A4 |
| TFEB |
| SELENON |
| GRM4 |
| SNX12 |
| ST8SIA2 |
| SIK3 |
| KMO |
| METTL11B |
| WHAMM |
| MASP1 |
| MYH4 |
| ZHX1 |
| IRF8 |
| TUBB8 |
| TIMM21 |
| SPHKAP |
| PREX1 |
| S100A7 |
| TCF23 |
| SEMA5A |
| MAK |
| PSMC3 |
| NOBOX |
| VGF |
| ACOT4 |
| TEKT5 |
| PTPRU |
| ATP8A2 |
| GSTM5 |
| COL10A1 |
| GOLGA8T |
| STMN4 |
| POMC |
| ZNF689 |
| CERCAM |
| SMIM10L2A |
| CPXCR1 |
| ZNF570 |
| SV2C |
| CNNM3 |
| ALAS2 |
| NCAN |
| ZNF2 |
| GMDS |
| FASTKD1 |
| AGAP1 |
| CD101 |
| SLC22A24 |
| FREM1 |
| IL20RB |
| SAA2 |
| ZNF592 |
| DNTT |
| TNFSF9 |
| HMCN2 |
| TACO1 |
| CNIH3 |
| GTF3C1 |
| NR1I2 |
| TREM1 |
| NXPE1 |
| SLC13A3 |
| SCN10A |
| SLIT2 |
| WDYHV1 |
| SEMA6B |
| GAGE12D |
| TTPA |
| LONRF1 |
| ERRFI1 |
| PHF10 |
| LOXHD1 |
| UHRF1 |
| PEX5L |
| ANKRD13D |
| PSAPL1 |
| OLA1 |
| ETV6 |
| KCNJ11 |
| CD300E |
| SEC24A |
| ARHGEF19 |
| ADGRV1 |
| PIGC |
| RELN |
| TTLL5 |
| SGK3 |
| SPTBN4 |
| TRPS1 |
| XIRP2 |
| KRBA1 |
| ZNF341 |
| MAJIN |
| ZSCAN22 |
| XKR4 |
| EPHA10 |
| STAB2 |
| KCNJ15 |
| MOCS2 |
| ADAM11 |
| USP28 |
| HCRT |
| NRG1 |
| ADAMTSL3 |
| STRN |
| SYT9 |
| EIPR1 |
| FAM81A |
| CHRNG |
| AR |
| MAPK4 |
| PLXDC1 |
| GLP2R |
| PHLDB2 |
| KIF3C |
| TMEM56 |
| MAP2K3 |
| FLT4 |
| FAM3D |
| SLC4A4 |
| C20orf96 |
| GLI4 |
| ZNF683 |
| RPL26L1 |
| PHTF2 |
| SMAP2 |
| CALCOCO1 |
| PZP |
| C2orf16 |
| ZBTB32 |
| SMG9 |
| HEYL |
| ADCY9 |
| FCRL1 |
| MEP1B |
| DTNA |
| PPP4R4 |
| PDYN |
| TMEM26 |
| DAPK1 |
| MAP3K19 |
| ATP10B |
| RELL2 |
| PASK |
| FOSL1 |
| NPC1 |
| SF3B3 |
| NDEL1 |
| COL6A6 |
| SPANXN3 |
| EVL |
| ARHGAP27 |
| KCNE4 |
| URM1 |
| CLN3 |
| MISP |
| MBD1 |
| TPD52L3 |
| ITGAL |
| CENPM |
| JHY |
| VAV1 |
| SCN5A |
| KAT6B |
| FBXO10 |
| ECSCR |
| FLVCR2 |
| RBL1 |
| FOLR3 |
| OR10A7 |
| VGLL4 |
| VRTN |
| AMER1 |
| KCP |
| ABLIM1 |
| IGF2BP1 |
| RBMS1 |
| PRSS1 |
| ZNF445 |
| TMEM54 |
| HNRNPA1P48 |
| C1QTNF8 |
| PGBD4 |
| NCR3LG1 |
| C9 |
| ALPK2 |
| CIART |
| ST18 |
| UBC |
| CD1C |
| BCL2L14 |
| CHRNA7 |
| MAGEA4 |
| C3orf67 |
| AVP |
| CCDC88A |
| FANCD2 |
| SPOCD1 |
| HECW1 |
| HMGB1 |
| FAM174B |
| DHRS2 |
| BICDL2 |
| RXFP2 |
| WDR63 |
| SPEF2 |
| HMSD |
| DNAJC10 |
| PLOD1 |
| ACTN3 |
| CUBN |
| MICU3 |
| CXCL14 |
| AL159163.1 |
| ZDHHC2 |
| APOBEC4 |
| HS3ST3A1 |
| PADI1 |
| NDUFA6 |
| SMDT1 |
| CHRM5 |
| KDELC2 |
| CACNG5 |
| GPR83 |
| AMBP |
| CD8B2 |
| SPAG11B |
| RNF223 |
| DDX53 |
| CLEC1B |
| FGF17 |
| ANKRD35 |
| A2ML1 |
| ROR2 |
| ZNF385B |
| ALDH2 |
| FRRS1L |
| GREB1 |
| MARK1 |
| C17orf99 |
| CYP21A2 |
| NLRP6 |
| ASCL5 |
| PIRT |
| SERPINE3 |
| AC104304.1 |
| AC105052.1 |
| TNXB |
| SERF1A |
| CRYBA4 |
| BAMBI |
| VSNL1 |
| SDC2 |
| TCHHL1 |
| FUT6 |
| FGF20 |
| ACADL |
| MYOCOS |
| MYOT |
| CDX2 |
| WDR88 |
| CHRNB2 |
| DBH |
| CROCC2 |
| TFAP2B |
| PRRT1B |
| C8orf86 |
| ANTXR2 |
| OSR2 |
| PRSS56 |
| ZP1 |
| C15orf59 |
| TUBA3E |
| LRRC31 |
| DUSP26 |
| C1orf21 |
| NDNF |
| CPB2 |
| FGF19 |
| PRRX1 |
| FAM189B |
| CCDC170 |
| ATP23 |
| PHF21B |
| PITX2 |
| SLC22A11 |
| LCN15 |
| ADGRG4 |
| ODAM |
| NDRG2 |
| FBLN1 |
| SMIM33 |
| EDAR |
| FRMD6 |
| EDN3 |
| VLDLR |
| CCDC158 |
| ASPA |
| EPS8L3 |
| KIT |
| POTEJ |
| HS6ST2 |
| SPATA22 |
| SCGB1D4 |
| FGF4 |
| KLK4 |
| TTLL2 |
| SPINK7 |
| TAS2R1 |
| SPAG11A |
| AC093668.2 |
| ZNF750 |
| KRT74 |
| KCNH6 |
| CCNJL |
| CRYGN |
| DEFA5 |
| ZNF705A |
| PRDM13 |
| BSX |
| ANG |
| UGT1A6 |
| ERICH3 |
| SLC16A11 |
| UBL4B |
| LRRC9 |
| KRT31 |
| KERA |
| FRMPD2 |
| OR1B1 |
| PRAC2 |
| IGSF5 |
| SERPIND1 |
| C20orf202 |
| CST5 |
| TSPAN5 |
| HIST1H4K |
| ZNF521 |
| FOXA3 |
| SERPINA4 |
| ONECUT3 |
| GALNT10 |
| RIPPLY2 |
| DMRTA2 |
| ANGPTL1 |
| CCDC129 |
| TMEM89 |
| B3GALT2 |
| CPA6 |
| DSG4 |
| GREM2 |
| SCTR |
| IHH |
| GLIS1 |
| CST4 |
| VCX3B |
| TCF7L1 |
| C9orf129 |
| HNF1A |
| EFCAB10 |
| FSD2 |
| SFRP5 |
| MIXL1 |
| SRARP |
| KRT27 |
| DWORF |
| SCARA5 |
| SCGB1D1 |
| ATP1A2 |
| TMEM95 |
| FAM198B |
| NXPE4 |
| TSKS |
| GLYATL3 |
| ASCL4 |
| OLFM4 |
| C20orf85 |
| DNMBP |
| STXBP6 |
| AC010255.3 |
| CDHR4 |
| SKOR1 |
| AL096711.2 |
| MTURN |
| MT4 |
| ITIH6 |
| SLC6A5 |
| CSGALNACT1 |
| HIST1H4D |
| HOXC4 |
| C9orf84 |
| CCDC65 |
| CALML3 |
| SORCS3 |
| LRRC49 |
| KRTAP12-4 |
| VHLL |
| MCOLN3 |
| FOXL2 |
| GNE |
| FAM109B |
| COL2A1 |
| NOX3 |
| APOD |
| SINHCAF |
| SLC30A10 |
| CCDC38 |
| PRR15 |
| WDR72 |
| NLRP11 |
| C21orf62 |
| ALDH1A1 |
| KLB |
| SMIM31 |
| BAG2 |
| CDC42EP5 |
| TBX3 |
| AC025283.2 |
| RASL11B |
| GPER1 |
| ZNF483 |
| GATA5 |
| ABCB4 |
| RGL2 |
| HNF4A |
| SOX14 |
| BEX1 |
| ABHD12B |
| DDX43 |
| SMIM6 |
| GDF5 |
| GUCY2F |
| METTL7A |
| GNG11 |
| C1orf105 |
| PGR |
| CYP17A1 |
| APCDD1 |
| XG |
| TRPM3 |
| CCL21 |
| RPTN |
| SEZ6L |
| CYP27C1 |
| FGL1 |
| SLC26A5 |
| CFAP77 |
| HNF1B |
| SPINK6 |
| HAAO |
| MYMX |
| KAAG1 |
| CD8B |
| CHRNA3 |
| SIM2 |
| RHOBTB3 |
| HOXC11 |
| CYP2B6 |
| RND2 |
| C4BPB |
| WDR86 |
| MUC13 |
| SP8 |
| C22orf15 |
| FOXD4 |
| SOBP |
| SPATC1L |
| CKMT2 |
| C10orf82 |
| MYLK3 |
| TUBA4B |
| OR3A2 |
| EMILIN3 |
| ABCC8 |
| IGFBPL1 |
| NRK |
| SULT2B1 |
| CA6 |
| BMP5 |
| SCG5 |
| USP44 |
| C2orf40 |
| SNAP91 |
| KLK3 |
| ANKRD34C |
| TMEM37 |
| CLIC6 |
| FMN2 |
| SLC5A8 |
| AC000093.1 |
| FAM216B |
| IQGAP2 |
| SLC6A2 |
| SALL1 |
| DEPDC7 |
| FXYD6 |
| BAHCC1 |
| CRYM |
| COL15A1 |
| MMP16 |
| PAQR8 |
| SMAD9 |
| CRHR2 |
| FAM169A |
| FAM181B |
| KCNJ9 |
| C8orf34 |
| HABP2 |
| FAM92B |
| VIL1 |
| KIAA2012 |
| SLC23A1 |
| CNTLN |
| APOH |
| SLC18A2 |
| OPRD1 |
| RDH12 |
| CACNA1S |
| ANKRD29 |
| DYNC1I1 |
| FAM189A2 |
| RAB30 |
| PGC |
| ELF5 |
| MLPH |
| DNAI2 |
| ERBB4 |
| KCNJ2 |
| RALYL |
| XPNPEP2 |
| TMEM130 |
| KPNA7 |
| BBOX1 |
| XDH |
| EDN1 |
| SLC5A11 |
| DHCR24 |
| CCNA1 |
| HPN |
| TAS2R60 |
| AIFM1 |
| SH2D6 |
| TMC4 |
| OR2B6 |
| KLK6 |
| SLC28A3 |
| KLHL14 |
| C15orf48 |
| SLC6A4 |
| KCNF1 |
| KCNE1 |
| ITLN1 |
| HLA-DRB5 |
| NXNL2 |
| SLC12A8 |
| LRRN2 |
| SYN3 |
| BTNL9 |
| BCAN |
| LHB |
| ITGA3 |
| SOX21 |
| DEFB1 |
| YBX2 |
| PIGZ |
| GLUD2 |
| SLC8A1 |
| CLDN16 |
| MEOX1 |
| SHISA3 |
| ZBTB22 |
| RTN4R |
| ITGB4 |
| GDF6 |
| KLK8 |
| CCNE1 |
| MS4A2 |
| GRB7 |
| MUC16 |
| GMPR |
| KIAA1211L |
| TGFBR3L |
| SYT13 |
| BOP1 |
| ADGRF1 |
| PRKCI |
| SOD3 |
| GPR75-ASB3 |
| RASAL1 |
| LAMP3 |
| TNFSF10 |
| SORT1 |
| FNDC1 |
| CD82 |
| MBOAT1 |
| TONSL |
| MUC1 |
| RASD2 |
| KLRG2 |
| NOVA2 |
| A3GALT2 |
| MAEL |
| OPN4 |
| SLCO3A1 |
| CCDC185 |
| S100A5 |
| ELF3 |
| BCAM |
| PCK2 |
| PAG1 |
| MMP15 |
| ASS1 |
| PDE4C |
| POTEG |
| TFR2 |
| GPR19 |
| HTR3A |
| OXTR |
| ARHGAP4 |
| ZNF467 |
| METRN |
| FOLR1 |
| PTGES |
| DAPP1 |
| ARL4C |
| MFAP5 |
| PQLC2L |
| CHI3L1 |
| VTCN1 |
| CIITA |
| CACNA1B |
| DSC2 |
| MT1M |
| PNOC |
| AC068987.5 |
| SLC9A3 |
| SCRG1 |
| CLDN7 |
| SEPT3 |
| KRT17 |
| ZFP36L2 |
| VGLL1 |
| NMNAT2 |
| DYTN |
| SH2D2A |
| FNDC11 |
| PGM2L1 |
| C11orf86 |
| HP |
| PRND |
| LAMB3 |
| KCNJ6 |
| ANKRD20A1 |
| RNF182 |
| CLDN9 |
| SLCO1B7 |
| EPS8L1 |
| NPTX2 |
| MYPN |
| HPR |
| CARD9 |
| TH |
| AC008397.2 |
| SAMD10 |
| INF2 |
| CRB2 |
| COL23A1 |
| WWC1 |
| SEPT12 |
| SLURP2 |
| EFNA1 |
| FST |
| SYCE3 |
| PHOX2A |
| ZNF726 |
| S100A14 |
| DLGAP1 |
| CCDC27 |
| AOC1 |
| LYPD8 |
| C5orf46 |
| GABRB2 |
| XKR7 |
| HELZ2 |
| TMPRSS4 |
| NKX2-8 |
| CAPG |
| RASSF10 |
| CCL20 |
| ST3GAL6 |
| SPP1 |
| LRRIQ3 |
| GPR141 |
| DAND5 |
| USH2A |
| OLR1 |
| H2AFB3 |
| TRIM27 |
| P2RY2 |
| GCM1 |
| FAM71A |
| ZNF217 |
| RGCC |
| EPHX4 |
| NOX4 |
| PPP1R16A |
| IL15 |
| PCDH15 |
| SST |
| ADAMTS20 |
| GIMD1 |
| SYNGR4 |
| CDH6 |
| MXRA5 |
| GRM1 |
| ASPHD1 |
| PTX3 |
| PDE3A |
| C3orf80 |
| CTCFL |
| SPATC1 |
| ACPP |
| VEGFA |
| ENPP2 |
| CXXC5 |
| ACKR2 |
| GRIN2A |
| GAST |
| PTGIR |
| MEP1A |
| SLC35F3 |
| TSPAN2 |
| POLQ |
| SLC30A2 |
| LAMA5 |
| KRT6A |
| OASL |
| DUSP10 |
| BLK |
| AC091980.2 |
| FUT8 |
| PRSS41 |
| KRT81 |
| NNMT |
| COL8A1 |
| EPHB1 |
| SLC2A1 |
| BCL2A1 |
| RGS1 |
| ISG15 |
| TEAD4 |
| PLPPR5 |
| UCP2 |
| BNC1 |
| FGFBP1 |
| ADGRG6 |
| ADGRG1 |
| LHX1 |
| EEF1A2 |
| FAM83A |
| KLRF1 |
| HSD3B7 |
| XPR1 |
| BPIFB4 |
| LYPD6B |
| LGALS2 |
| L1CAM |
| DNAJB13 |
| SDS |
| PDZK1IP1 |
| INAVA |
| BHLHA9 |
| COL9A1 |
| C10orf55 |
| KRT14 |
| EPPK1 |
| RHPN1 |
| SYNDIG1 |
| ALOXE3 |
| IFNE |
| OSCAR |
| OR56A3 |
| MYH14 |
| CCR8 |
| IMPG2 |
| EMB |
| CRABP2 |
| PCDH1 |
| VWDE |
| KRT80 |
| AMY1A |
| CHP2 |
| ALOX15B |
| NUPR2 |
| ANGPTL4 |
| APELA |
| MAP3K21 |
| MMP8 |
| FAM83B |
| GFOD1 |
| ELFN2 |
| SMKR1 |
| S100A4 |
| CT45A10 |
| AGRN |
| CYP2S1 |
| IGF2 |
| LILRB1 |
| PDLIM1 |
| KLK7 |
| HIST1H1T |
| P2RY6 |
| SLC5A5 |
| CADM3 |
| LILRB3 |
| DNAJC5G |
| AK4 |
| NKAIN4 |
| GUCY1A2 |
| CLDN3 |
| KLK13 |
| IZUMO1 |
| ADAMTS15 |
| PTPRQ |
| PGA3 |
| ODF3L1 |
| SLAMF9 |
| FXYD5 |
| ERFE |
| CHI3L2 |
| C17orf64 |
| ADORA1 |
| PLEKHN1 |
| CSMD2 |
| CLCN1 |
| KIFC2 |
| NECTIN4 |
| AC008764.1 |
| ROPN1B |
| SLC12A1 |
| WNT7A |
| RNF152 |
| TBC1D10C |
| GRM5 |
| NEU4 |
| ANKRD62 |
| TFAP2C |
| CD74 |
| KRT16 |
| METRNL |
| CX3CL1 |
| FBLN2 |
| ARAP2 |
| GPR50 |
| C1orf167 |
| KLK5 |
| NRG3 |
| EFNB2 |
| ICAM1 |
| COMP |
| TRIM54 |
| THBS1 |
| ANKRD2 |
| SHROOM3 |
| CELSR1 |
| DAXX |
| LRRC7 |
| HIST1H4F |
| CXCL1 |
| SYT8 |
| PAX8 |
| TMEM74B |
| HSH2D |
| BHLHE40 |
| WISP1 |
| KCNIP4 |
| CETP |
| C4orf51 |
| GRIK3 |
| WSCD2 |
| KRT7 |
| SALL4 |
| TMC3 |
| NOTCH4 |
| RHBDL2 |
| IL7R |
| PQLC1 |
| CLVS1 |
| GIPC3 |
| GDPD2 |
| SPATA21 |
| TM4SF1 |
| PCDHB1 |
| MSLN |
| FLG |
| ALS2CL |
| CLDN20 |
| HLA-DRB1 |
| TNFAIP2 |
| BTBD19 |
| NFKBIE |
| KLK10 |
| IL32 |
| PPP2R2B |
| TMEM59L |
| LAMC2 |
| HS3ST2 |
| SPSB1 |
| CLEC5A |
| SCNN1A |
| PPP1R1C |
| CELSR2 |
| GABRA5 |
| SLC34A2 |
| ZBED2 |
| FOXH1 |
| FAM107A |
| NMU |
| EGLN3 |
| SLURP1 |
| MICALL2 |
| BCL3 |
| ITGB3 |
| CLRN3 |
| ANKRD20A8P |
| CPE |
| SYCE2 |
| BSND |
| NLRP12 |
| S100A7A |
| LCN2 |
| FAM57B |
| RSPO3 |
| SBK2 |
| LY6D |
| RIPOR2 |
| DDR1 |
| PTK6 |
| PTGS1 |
| COL11A1 |
| DOCK3 |
| SUCNR1 |
| OLFM3 |
| CBLC |
| KRTAP5-10 |
| L1TD1 |
| BCAT1 |
| AHNAK2 |
| CARMIL2 |
| PHOSPHO1 |
| RECQL4 |
| GOLGA6A |
| MMP19 |
| HMGA1 |
| SERPINA3 |
| TM4SF19 |
| NAA11 |
| CYP4F11 |
| STMN2 |
| KCNJ16 |
| AC007906.2 |
| CXCL16 |
| MMP10 |
| PPP2R3A |
| SLC4A11 |
| B4GALNT2 |
| TAS2R50 |
| SFXN5 |
| CLCF1 |
| E2F3 |
| TMEM215 |
| DEPP1 |
| KRTAP5-7 |
| DENND2D |
| SLC52A2 |
| CP |
| BPIFB3 |
| DNAH5 |
| STK17B |
| ATP8B1 |
| TTC16 |
| ADD2 |
| MAL2 |
| PARD6B |
| HCAR1 |
| SCN9A |
| HTRA4 |
| CYP1B1 |
| CYP4B1 |
| GRIA2 |
| ROS1 |
| SLC5A4 |
| ABCA13 |
| EYA2 |
| HAPLN1 |
| TNNT1 |
| GDF3 |
| MMP9 |
| BAIAP2L1 |
| NPDC1 |
| ERGIC1 |
| MCTS1 |
| TPSAB1 |
| PRDM11 |
| CACNA1D |
| WNK2 |
| NDUFV3 |
| PGAM5 |
| NAP1L2 |
| ATP1A3 |
| LILRB2 |
| CLEC3A |
| CLDN15 |
| MTNR1B |
| TELO2 |
| CKMT1B |
| WSCD1 |
| KNDC1 |
| F13A1 |
| SLC9A5 |
| CRX |
| HRH3 |
| C20orf27 |
| UROC1 |
| GGT2 |
| IRAK2 |
| SLC44A2 |
| SAG |
| CTRC |
| RNF126 |
| RAC2 |
| SSX2 |
| KCND1 |
| SLC8A2 |
| FAM136A |
| TACC3 |
| SCX |
| NDUFA11 |
| SLC15A1 |
| OSGIN1 |
| DNAH17 |
| NKD1 |
| RBM33 |
| GSTT2B |
| RIMBP3B |
| GPAT4 |
| NAT8L |
| CCDC57 |
| WDR59 |
| PRIMA1 |
| BCR |
| PUS3 |
| ANKRD20A3 |
| CORO2A |
| IRGM |
| PLA2G2D |
| WNT2 |
| ADAMTS8 |
| EPB42 |
| SMARCA4 |
| SCIN |
| SSH1 |
| NUTM2D |
| ULK1 |
| CAMK2A |
| RNF166 |
| QRICH2 |
| KIRREL2 |
| CCDC33 |
| CHFR |
| CLPTM1 |
| SLC39A13 |
| STPG1 |
| BDKRB2 |
| CA10 |
| NUDC |
| PAOX |
| SYN1 |
| LETM1 |
| SRPK3 |
| STRBP |
| CLP1 |
| GDI1 |
| RASIP1 |
| ARHGEF2 |
| MICAL3 |
| FIGNL2 |
| CYP11B2 |
| ELSPBP1 |
| PIWIL2 |
| POU4F1 |
| ISG20 |
| KDM4B |
| NOP14 |
| PAK5 |
| HRH1 |
| COX10 |
| EHMT1 |
| ZNF79 |
| IKBKE |
| LHX5 |
| DIDO1 |
| AP3D1 |
| NPHS1 |
| RBM19 |
| CHST12 |
| SLC9A4 |
| PTPRN |
| PADI2 |
| DNAH10 |
| VASN |
| CASR |
| RTN4RL1 |
| RUNX2 |
| ASH1L |
| XCR1 |
| SLC35E2 |
| PCDHA11 |
| PLXNB3 |
| DYRK2 |
| ZNF275 |
| PCDHGC5 |
| IFITM10 |
| ARHGEF1 |
| HIP1R |
| FSTL4 |
| MMP23B |
| ATP7B |
| PCDH9 |
| CEP131 |
| TCF3 |
| CCDC88C |
| KIF1A |
| POLRMT |
| DOCK5 |
| KIF25 |
| IGHD |
| TNFRSF4 |
| CUL9 |
| SEPT8 |
| RGS12 |
| CR2 |
| SH3BGRL3 |
| KRTDAP |
| POTEI |
| APBA2 |
| LMTK3 |
| MARK4 |
| FOXN1 |
| FOXD4L6 |
| SLC51B |
| VWCE |
| SARDH |
| PADI6 |
| SH2D4B |
| ARRDC5 |
| TANC2 |
| C1orf100 |
| ELN |
| TFE3 |
| ANXA8 |
| KLHL26 |
| MMP25 |
| LRP5L |
| CHST5 |
| KCNB1 |
| ACACB |
| SHISAL1 |
| DOC2B |
| TPCN2 |
| FPR2 |
| HS3ST6 |
| MIP |
| MAD1L1 |
| ZFHX3 |
| TMEM145 |
| TRPV3 |
| ITPR1 |
| RNF224 |
| MANSC1 |
| CUX1 |
| WDR90 |
| MUC5AC |
| ADAMTSL4 |
| KSR2 |
| PLB1 |
| SSC5D |
| MAP4K3 |
| DLGAP2 |
| TREML4 |
| ADGRA1 |
| MRC2 |
| NEURL1B |
| DCHS1 |
| CDKL5 |
| LINGO1 |
| SLC35B1 |
| DKK4 |
| EXOC3L1 |
| LAMC3 |
| SPTBN5 |
| CATSPERD |
| SLC4A2 |
| FAM181A |
| SOST |
| ARHGEF18 |
| RFX4 |
| SCAMP5 |
| CMKLR1 |
| HOXA6 |
| HSF5 |
| DGKQ |
| ZNF579 |
| SDK2 |
| BAIAP3 |
| NOD2 |
| ALS2CR12 |
| EPHB3 |
| SNCA |
| PLPP2 |
| TSPYL5 |
| ZFHX2 |
| ICOSLG |
| RGR |
| PCNX3 |
| CDR2L |
| C13orf46 |
| PPP1R26 |
| CRLF2 |
| TUBB3 |
| PIK3CD |
| ACAN |
| GRAMD4 |
| ATG9A |
| MEX3C |
| BRD3 |
| ATCAY |
| SPERT |
| THEG |
| TNPO2 |
| MALL |
| C22orf23 |
| TMEM132A |
| TECPR1 |
| MYO18B |
| EPN1 |
| LGALS14 |
| PIEZO1 |
| PCSK9 |
| RAVER1 |
| ARHGAP23 |
| LRPAP1 |
| OCA2 |
| CTCF |
| OTOG |
| CLCNKB |
| INPP5A |
| BAIAP2 |
| FILIP1 |
| SYNGR1 |
| MYBPC2 |
| CDK3 |
| SLIT1 |
| NCLN |
| MEGF6 |
| REEP1 |
| VCX2 |
| CDK10 |
| KCNJ14 |
| SLC25A53 |
| ZNF316 |
| RNF213 |
| CDHR2 |
| FGFRL1 |
| TGM6 |
