# Supplementary material for: Contribution of m5C RNA Modification-Related Genes to Prognosis and Immunotherapy Prediction in Patients with Ovarian Cancer
Source: Mediators Inflamm. 2023 Nov 13;2023:1400267. doi: 10.1155/2023/1400267 (PMC10661868; doi:10.1155/2023/1400267)
Supplement: Supplementary 2 — The forest plot of m5C RNA modification-related genes correlated with survival time. [file 1400267.f2.pdf]

	pvalue	Hazard ratio
TRIM27	0.004	0.676(0.517−0.882)
ARHGAP23	0.007	1.242(1.062−1.453)
FCGBP	<0.001	1.264(1.133−1.410)
OPA3	0.002	1.744(1.231−2.472)
USP14	0.009	0.688(0.520−0.911)
CRHR1	0.005	1.396(1.106−1.762)
ANGPTL4	0.004	1.206(1.062−1.370)
ZNF316	0.005	1.326(1.087−1.616)
RPL26L1	0.008	0.779(0.647−0.937)
HTRA4	0.003	0.261(0.108−0.631)
ZHX3	0.004	1.574(1.157−2.141)
ATP1A3	0.001	1.215(1.081−1.366)
CXCR2	<0.001	1.993(1.376−2.886)
GMPR	<0.001	0.813(0.727−0.909)
SLAMF1	<0.001	0.382(0.220−0.661)
CLEC5A	0.007	1.291(1.071−1.556)
GRIN2D	0.004	1.267(1.078−1.489)
ZFHX4	<0.001	1.925(1.382−2.680)
PYGB	0.003	1.414(1.127−1.775)
CUX1	0.004	1.619(1.163−2.255)
ISG20	<0.001	0.695(0.569−0.848)
PLA2G2D	<0.001	0.661(0.522−0.836)
CD38	<0.001	0.613(0.476−0.791)
AGAP1	0.003	1.487(1.142−1.936)
SRL	0.009	1.501(1.105−2.040)
GALNT10	<0.001	1.681(1.281−2.205)
LAMP3	0.004	0.837(0.741−0.945)
BAG2	0.009	0.772(0.636−0.937)
ALDH5A1	0.009	0.752(0.608−0.930)
SUB1	0.008	0.751(0.608−0.927)
GRAMD4	<0.001	1.599(1.212−2.109)
FOXK1	0.006	1.409(1.106−1.796)
PTPRU	0.004	1.243(1.072−1.440)
SHISAL1	0.009	1.377(1.084−1.749)
SORCS2	0.002	1.309(1.107−1.547)
EXOSC3	0.003	0.635(0.470−0.858)
HMBS	0.009	0.707(0.545−0.916)
GPAT4	0.004	1.561(1.154−2.113)
GALNT6	0.008	0.789(0.662−0.940)
NDUFV2	0.003	0.721(0.581−0.895)
NUGGC	0.002	0.413(0.237−0.721)
AHNAK2	0.008	1.205(1.050−1.382)
FKBP3	0.007	0.732(0.585−0.917)
KLK4	0.006	1.905(1.203−3.018)
ALDOC	0.009	1.177(1.042−1.330)
PIEZO1	0.002	1.289(1.098−1.514)
MAP3K4	0.002	1.764(1.237−2.513)
HMCN2	<0.001	1.425(1.177−1.725)
SYDE1	0.003	1.319(1.099−1.583)
MAP3K13	0.001	0.698(0.563−0.866)
ERBB2	0.008	1.291(1.070−1.558)
PRSS1	0.006	0.867(0.783−0.959)

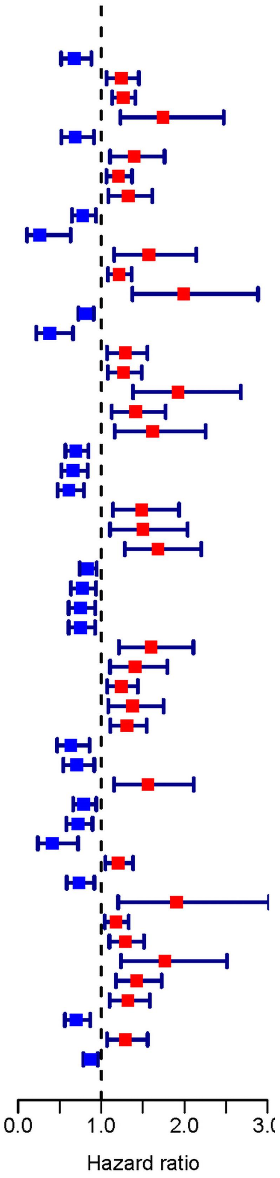

Additional file 2: Figure S1. The forest plot of m5C RNA modification-related genes correlated with survival time.
