# Supplementary material for: Contribution of m5C RNA Modification-Related Genes to Prognosis and Immunotherapy Prediction in Patients with Ovarian Cancer
Source: Mediators Inflamm. 2023 Nov 13;2023:1400267. doi: 10.1155/2023/1400267 (PMC10661868; doi:10.1155/2023/1400267)
Supplement: Supplementary 3 — The slightly positive correlation between PLA2G2D and m5C RNA modification regulators. [file 1400267.f3.pdf]

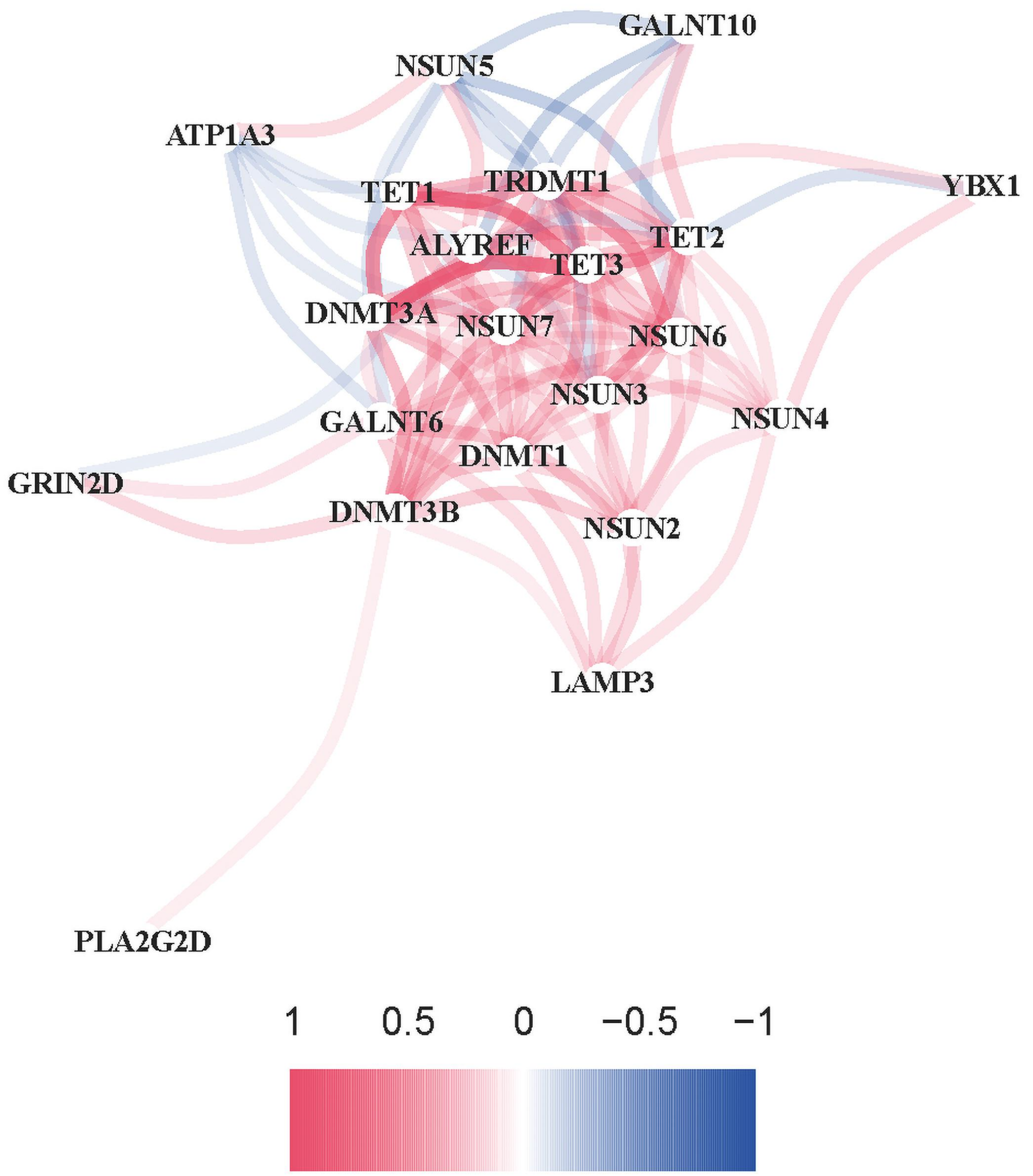

Additional file 3: Figure S2. The slightly positive correlation between PLA2G2D and m5C RNA modification regulators.
