# Supplementary material for: Contribution of m5C RNA Modification-Related Genes to Prognosis and Immunotherapy Prediction in Patients with Ovarian Cancer
Source: Mediators Inflamm. 2023 Nov 13;2023:1400267. doi: 10.1155/2023/1400267 (PMC10661868; doi:10.1155/2023/1400267)
Supplement: Supplementary 4 — The DEGs were used for functional enrichment analysis. [file 1400267.f4.docx]

Table S2. The DEGs used for functional enrichment analyses.

| Gene |
| --- |
| TCF21 |
| IGFL2-AS1 |
| IGHV3-33 |
| AL357507.1 |
| NCCRP1 |
| CD38 |
| IGLV3-25 |
| CCL8 |
| ATP1A3 |
| IGHGP |
| MZB1 |
| VSTM2L |
| LINC02167 |
| UBD |
| COL11A1 |
| LRRC15 |
| SERPINE1 |
| COX8C |
| MMP12 |
| CD79A |
| GZMB |
| IGHM |
| CXCL11 |
| CCL18 |
| IGKV4-1 |
| CCL13 |
| AL589182.1 |
| SLAMF7 |
| SLURP1 |
| IGKJ5 |
| MEIS3 |
| AC006262.4 |
| SIX3 |
| CCL19 |
| IGHV3-64 |
| OR7E22P |
| COL5A1 |
| JCHAIN |
| LINC02506 |
| IGLV3-19 |
| AADACP1 |
| UGT2B17 |
| IGHG1 |
| CXCL9 |
| OGN |
| IGLV9-49 |
| PSLNR |
| FABP4 |
| CD3D |
| IGKV1-17 |
| CRYGB |
| CCL7 |
| LINC02303 |
| IGKV5-2 |
| IGKV3-20 |
| CXCR2P1 |
| KIF26B |
| NGFR |
| HLA-G |
| KRT6A |
| ACTG2 |
| IGKV3-15 |
| OR2I1P |
| IGLC3 |
| IGKV2-24 |
| SFTA2 |
| CXCL13 |
| CXCL10 |
| IGKV2-30 |
| ADAMDEC1 |
| FOXD3-AS1 |
| PYY |
| PRSS2 |
| DPEP3 |
| IGKC |
| GRIN2D |
| PRSS1 |
| KRT4 |
| IGLC2 |
| IFI6 |
| LINC02683 |
| IGKV1-8 |
